# Histone variant innovation in a rapidly evolving chordate lineage

**DOI:** 10.1186/1471-2148-11-208

**Published:** 2011-07-15

**Authors:** Alexandra Moosmann, Coen Campsteijn, Pascal WTC Jansen, Carole Nasrallah, Martina Raasholm, Henk G Stunnenberg, Eric M Thompson

**Affiliations:** 1 Sars International Centre for Marine Molecular Biology, Norway; 2 Department of Biology University of Bergen, Norway; 3 Department of Molecular Biology, Nijmegen Center for Molecular Life Sciences, Radboud University Nijmegen, The Netherlands; 4 Centre for Cancer Biomedicine, Faculty of Medicine, University of Oslo, and Department of Biochemistry, Institute for Cancer Research, Norwegian Radium Hospital, Oslo University Hospital, Oslo, Norway; 5 Department of Molecular Cancer Research, University Medical Center Utrecht, Utrecht, The Netherlands

**Keywords:** histone complement, DNA repair, urochordate, posttranslational modification, endocycle, gametogenesis, testes, H2A.Z, alternative splicing

## Abstract

**Background:**

Histone variants alter the composition of nucleosomes and play crucial roles in transcription, chromosome segregation, DNA repair, and sperm compaction. Modification of metazoan histone variant lineages occurs on a background of genome architecture that shows global similarities from sponges to vertebrates, but the urochordate, *Oikopleura dioica*, a member of the sister group to vertebrates, exhibits profound modification of this ancestral architecture.

**Results:**

We show that a histone complement of 47 gene loci encodes 31 histone variants, grouped in distinct sets of developmental expression profiles throughout the life cycle. A particularly diverse array of 15 male-specific histone variants was uncovered, including a testes-specific H4t, the first metazoan H4 sequence variant reported. Universal histone variants H3.3, CenH3, and H2A.Z are present but *O. dioica *lacks homologs of macroH2A and H2AX. The genome encodes many H2A and H2B variants and the repertoire of H2A.Z isoforms is expanded through alternative splicing, incrementally regulating the number of acetylatable lysine residues in the functionally important N-terminal "charge patch". Mass spectrometry identified 40 acetylation, methylation and ubiquitylation posttranslational modifications (PTMs) and showed that hallmark PTMs of "active" and "repressive" chromatin were present in *O. dioica*. No obvious reduction in silent heterochromatic marks was observed despite high gene density in this extraordinarily compacted chordate genome.

**Conclusions:**

These results show that histone gene complements and their organization differ considerably even over modest phylogenetic distances. Substantial innovation among all core and linker histone variants has evolved in concert with adaptation of specific life history traits in this rapidly evolving chordate lineage.

## Background

In eukaryotes, chromatin is the interface through which genetically encoded information is read to orchestrate a diversity of cellular and organismal functions. Chromosomal packaging of nuclear DNA is achieved through small basic histone proteins. The highly conserved core histones, H4, H3, H2A and H2B form an octamer of two H2A/H2B dimers flanking a central H3/H4 tetramer that wrap ~147 bp of DNA in 1.7 turns to form the nucleosome core particle (NCP) [1]. Tetramer formation is common to archaeal and eukaryotic histones. The eukaryotic origin of H2A-H2B dimers, doubling the DNA wrap capacity, has been discussed in light of demands of increased genome size [2] and kinetic constraints of DNA compaction imposed by eukaryotic mitosis [3]. Interactions between the histone octamer and DNA are further modulated by linker histones H1/H5, which associate with DNA at its entry/exit site on the NCP surface [4]. Roles of histones in structural compaction must be balanced with regulatory mechanisms that permit selective access to DNA to enable functions such as transcription, replication and DNA repair. Three major strategies contribute to regulatory remodeling of chromatin: ATP-dependent complexes that act on nucleosomes to modify accessibility of wrapped DNA sequences to trans-acting factors [5], the deployment of histone variants that alter nucleosome dynamics, and covalent posttranslational histone modifications (PTMs) including acetylation, methylation, phosphorylation, ubiquitylation, citrullination, ADP-ribosylation, glycoslylation and sumoylation [6,7]. One current view is that eukaryotic genomes are indexed locally, and over broader regions, through a combination of histone variants and their diverse PTMs [8,9].

Main lineages of canonical core histones, constituting the bulk of histone proteins, are assembled into chromatin during DNA replication. These replication dependent (RD) genes lack introns, are typically organized in gene clusters and their mRNAs possess a conserved stem-loop (SL) in the 3'UTR coupling gene expression to DNA replication. In contrast, histone variants are often transcribed from orphan genes that contain introns, lack the SL and their expression is not restricted to S-phase. Consequently, they are referred to as replacement or replication-independent (RI) variants. From an evolutionary perspective, histone variants are classified as universal or lineage-specific variants, where the term variant refers to non-allelic sequence variation. Universal variants have ancient functions common to eukaryotic cells, whereas lineage-specific variants appear specialized to certain organismal requirements. Core histones clearly differ in their evolutionary propensity to diversify as distinct variants. H4 is highly constrained as it makes contacts with the other 3 core histones and its N-terminal tail residues are subject to extensive PTMs. This is reflected in its nearly invariant stature. No H4 amino acid sequence variants have been reported in any multicellular organism to date and diversified H4s are known only in trypanosomes and ciliates [10,11]. Despite H2Bs showing substantially less evolutionary sequence constraint than H3 and H4 they, like H4, exhibit little specialization [12], with the exception of gametogenesis variants. On the other hand, H3 and H2A are much richer in their genealogy with diversification of a number of functionally essential variants.

The two H3 molecules interact with each other in the nucleosome, as do the two H2A molecules, whereas H4 and H2B do not. Two-fold nucleosomal symmetry is organized along the C-terminal four-helix bundle dimerization interface of the two H3s [1]. Interaction between the two H2As is mediated by their loop 1 (*L1*) domains. Specializations have evolved in the H3 lineage with at least two H3 variants present in most eukaryotes. The replacement variant H3.3 can undergo RD or RI assembly and deposition is primarily in transcribed regions of euchromatin [13,14]. H3.3 differs from canonical H3 at few positions, predominantly, one in the N-terminal tail and three in the α2 helix of the histone fold domain (HFD), the latter three required for RI assembly [13]. All eukaryotes possess H3.3 though Asomycetes lack canonical H3. Phylogenetically, the two proteins appear not to be separate, early-branching lineages, as distinct H3 and H3.3 histones have arisen multiple times [3]. A second common H3 variant is centromeric H3 (CenH3) essential for recruitment of kinetochore components vital to mitosis. CenH3s retain only ~50% identity to H3s in the HFD and have divergent N-terminal tails from 20 to 200 residues. CenH3 phylogeny is poorly resolved. They appear to have arisen many times with rapid divergence under positive selection. There are also lineage-specific meiotic and spermatogenesis H3 variants [15].

Among core histones, H2As have evolved the greatest variant diversity. There are two nearly universal variants, H2AX and H2A.Z. H2AX plays an important role in the maintenance of eukaryotic genome integrity by participating in double-strand DNA-break (DSB) repair by nonhomologous end joining (NHEJ). H2AX has arisen multiple times during evolution but similar constraints have led to convergent acquisition of the H2AX-specific phosphorylation motif SQE/DΦ (Φ = hydrophobic residue) [3]. Phosphorylation of the S residue in this C-terminal motif forms γH2AX, which assists in the recruitment of histone modifiers, chromatin remodelers and DNA repair complexes. H2AX has entirely replaced canonical H2A in fungi and giardia [3,16] but is absent in *C. elegans *and protozoan parasites, *Plasmodium *and trypanosomes. H2A.Z had a single evolutionary origin, and has remained distinct from canonical H2A [17]. It is essential for viability in a range of species, [18-20] and is implicated in gene activation, chromosome segregation, heterochromatin silencing, and cell cycle progression [21]. A common thread in a number of these disparate roles may be the proclivity of H2A.Z to form more stably positioned nucleosomes [18] combined with differential use of PTMs. Other H2A variants include the C-terminally extended macroH2A, the C-terminally truncated mammalian H2A.Bbd and an array of more poorly studied lineage-specific variants.

Linker H1 variants form a complex family and subtypes are classified as canonical RD (H1.1-H1.5 in human) or RI linker histones (H1x and H1.0 in human). They display tissue and developmental specificity, with vertebrates commonly expressing testes-specific (H1t, H1T2, Hils1) and oocyte-specific (H1oo) variants [22]. Structurally, metazoan H1s are divided into three domains: a short, flexible N-terminal, a globular domain containing a winged-helix fold and a long, lysine rich C-terminal tail. H1 variants differ in biophysical properties, association with repressed or active chromatin and their ability to increase or decrease transcription when over-expressed.

The above picture is derived from histone sequence comparisons in a wide array of eukaryotes, but to date, very few full histone complements have been analyzed more comprehensively and information is principally restricted to universal variants as well as some lineage-specific variants in mammals and plants. Modification of metazoan histone variant lineages occurs on a background of genome architecture that shows global similarities from sponges to vertebrates [23] but the urochordate, *Oikopleura dioica *(Od), a member of the closest sister group to vertebrates, demonstrates that this ancestral genome architecture can be profoundly modified [24]. In this fast-evolving, pan-global zooplankton, a repertoire of >18000 genes has been extraordinarily compacted into a genome of only 70 Mb, with sizes of introns and intergenic regulatory regions greatly reduced. Whereas other chordates generally grow through cell proliferation, rapid growth of *O. dioica *over a short, 6-day life cycle is achieved principally through endoreduplication [25]. Here, we characterize the highly diverse histone complement that has emerged in concert with evolution of this intriguing lineage at the invertebrate-vertebrate transition.

## Results

### Histone gene organization in *Oikopleura dioica*

**Figure 1:**
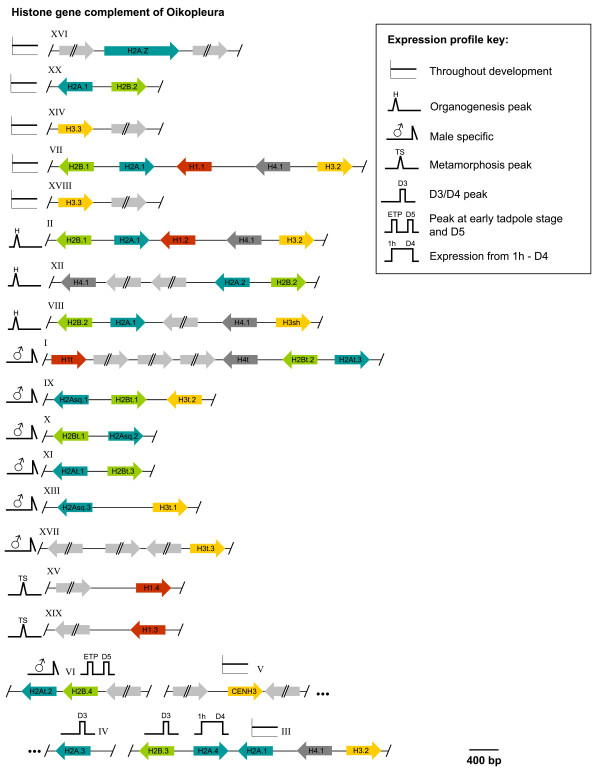
***O. dioica *histone gene complement**. Histone clusters consist of 2-5 genes dispersed throughout the genome (loci given as roman numerals). Genes belonging to a given cluster were transcriptionally co-regulated and developmental expression profiles of the clusters are indicated with icons (key: H, hatching; ETP, early tadpole; TS, tailshift; D3/4/5, day 3/4/5). Expression of histone genes is mostly driven from short bidirectional promoters, with the exception of several replacement variants (H3.3, H2A.Z, H1.4, H1.3 and CenH3) dispersed as orphan genes. Expression was undetectable for a truncated H3 (H3sh, cluster VIII), likely to be a pseudogene. Light grey arrows indicate non-histone genes.

Histone genes were dispersed throughout the *O. dioica *(Od) genome with most in clusters up to quintets. In total, 47 histone genes (6 H4, 10 H3, 15 H2A, 11 H2B and 5 H1 genes) encoding 31 different histone proteins (2 H4, 6 H3, 11 H2A, 7 H2B and 5 H1) were identified (Figure 1). Clustered genes showed typical RD features. They were intron-less and most shared divergent promoters. Primary transcripts of these genes contain both a conserved SL and a downstream polyadenylation (polyA) signal in their 3' UTRs but lack the histone downstream element [26,27]. A second set of histone genes were present as orphan genes: 3 H3 (H3.3, CenH3 and H3t.3), 2 H1 (H1.3 and H1.4) and 2 H2A isoforms (H2A.3 and H2A.Z). These genes have introns (except H2A.3 and H3t.3) and the SL is degenerate or absent (Additional File 1, Table S1), suggesting they are RI variants. To assess phylogeny among histone families, unrooted maximum likelihood trees were generated. As expected, genes encoding the same histone protein were more closely related and the most divergent histones exhibited the longest branch lengths (Additional File 1, Fig. S1). Clusters I, IX and XI, had the same H2A and H2B phylogenies and likely result from more recent gene duplications.

To compare the *O. dioica *histone complement to that of other urochordates we extended genome searches to the histone genes of two ascidians, *Ciona intestinalis *and *Ciona savignyi*. Surprisingly, we found that the histone complements of the two ascidians differed significantly in terms of histone gene number and organization. The *C. savignyi *genome (~180 Mb) contains a high number (~130) of histone genes partly organized in large clusters, whereas the *C. intestinalis *genome (~156 Mb) encoded only 27 histone genes, mostly interspersed among non-histone genes. A locus within the *C. savignyi *genome was found where histone genes are arranged similarly to the tandem-arrays of sea urchin and *Drosophila*. Table 1 shows that histone gene complements and their organization can differ considerably even over modest phylogenetic distances.

**Table 1:** Histone gene variants and their genomic organization from yeast to vertebrates.

	*M. musculus/H. Sapiens*	*O. dioica*	*C. intestinalis*	*C. savignyi*	*S. purpuratus*	*D. melanogaster*	*C. elegans*	*S. cerevisiae*
**Genomic organization**	Large non-tandem clusters	Small, dispersed gene groups	Few dispersed genes	Large, mostly non-tandem clusters	Large tandem array clusters	Large tandem array clusters	Small, dispersed gene groups	Few dispersed genes

**H4 variants**								
H4.1	H4.1	H4.1	H4.1	H4.1	H4.1	H4.1	H4.1	H4.1
H4t (testis)	-	H4t	-	nd	-	-	-	-
+ isoforms^§^	-	-	-	yes	-	-	-	-

**H3 variants**								
H3.1	H3.1	-	-	-	-	-	-	-
H3.2	H3.2	H3.2	H3.2	H3.2	H3.2	H3.2	H3.2	-
H3.3	H3.3	H3.3	H3.3	H3.3	H3.3	H3.3	H3.3	H3.3
CenH3	CENP-A	CenH3	CenH3	CenH3	H3-Cid	CID	HCP-3	CSE4
H3t (testis)	H3t	H3t.1 H3t.2 H3t.3	-	nd	nd		nd	-
+ isoforms^§^	yes	-	-	yes	yes	-	yes	-

**H2B variants**								
H2B.1	H2B.1	H2B.1, H2B.2, H2B.3*^1^	H2B.1	H2B.1	H2B.1	H2B.1	H2B.1	Htb1p, Htb2p
H2Bt (testis)	TSH2B H2BFWT spH2B	H2B.4*^2^ H2Bt.1 H2Bt.2 H2Bt.3	nd	nd	SpermH2B	-	nd	-
+ isoforms^§^	yes	-	yes	yes	yes	-	yes	-

**H2A variants**								
H2A.1	H2A.1	H2A.1, H2A.2	H2A.1	H2A.1	H2A.1	H2A.1	H2A.1	-
H2A.Z	H2A.Z.1 H2A.Z.2	H2A.Z	H2A.Z	H2A.Z	H2A.Z	H2Av	HTZ-1	Htz1p
H2AX	H2AX	-	H2AX	H2AX	H2AX	H2Av	-	Hta2p
MacroH2A.1	MacroH2A.1	-	-	-	MacroH2A.1	-	-	-
MacroH2A.2	MacroH2A.2	-	-	-	MacroH2A.2	-	-	-
H2At (testis)	H2AL1 H2AL2	H2At.1 H2At.2 H2At.3 H2Asq.1 H2Asq.2 H2Asq.3	nd	nd	-	nd	nd	-
H2A.Bbd	H2A.Bbd	-	-	-	-	-	-	-
+ isoforms^§^	yes	H2A.3*^1^ H2A.4*^3^	yes	yes	yes	yes	yes	-

**H1 variants**								
H1.1	H1.1 H1.2 H1.3 H1.4 H1.5	H1.1 H1.2*^4^ H1.3*^5^ H1.4*^5^	3 isoforms	11 isoforms	H1 H1a H1b	H1	8 isoforms	Hho1p
H1t (testis)	H1t H1T2 H1LS1	H1t			SpermH1			
H1.oo (oocyte)	H1oo							
H1.0 (diff)^†^	H1.0 H1.x				H1.0			

^§^+ isoforms = additional isoforms in the genome. ^†^(diff) = differentiation H1 variant. Expression: peaks at *^1^D3/D4; *^2 ^peaks in early tadpoles and D5, where it is enriched in the testes; *^3^commences 1 h pf and decreases after D4; *^4 ^peaks during organogenesis and *^5^peaks at metamorphosis. The nomenclature of OdH1s does not imply direct orthology with human H1.1-H1.4

### Expression of histone gene clusters is developmentally co-regulated

**Figure 2:**
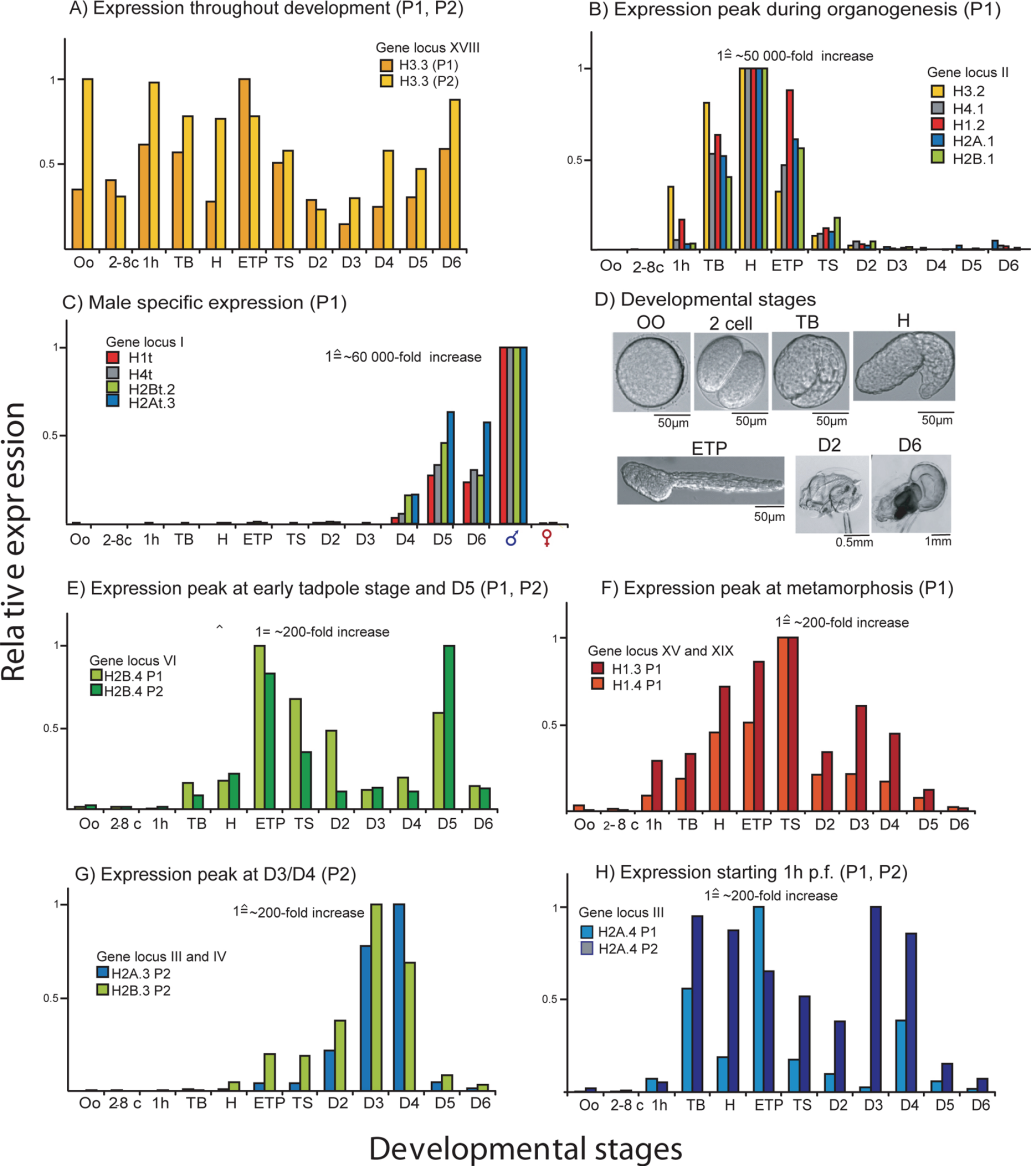
***O. dioica *histone gene expression profiles throughout development**. qRT-PCR of histone genes from 12 developmental stages (see D): oo (oocytes), 2-8c (2-8 cells), 1h (1h postfertilization (pf)), TB (tailbud, 2.5 h pf), H (hatched, 3 h pf), ETP (early tadpole 5-7 h pf), TS (tailshift metamorphosis, 9-12 h pf), D2/3/4/5/6 (Day 2/3/4/5/6). Expression patterns were classed in 7 profiles. Determinations were performed on two technical replicates on each of two biological population replicates (P1, P2). Histone genes within a cluster were transcriptionally co-regulated and had the same developmental profiles (A, C). A) The majority of canonical histone genes and several replacement variants such as H3.3 were expressed throughout development. B) All canonical histones had additional gene loci exclusively expressed during organogenesis. C) Male-specific expression of the histone gene cluster at locus I. Male- (♂) and female (♀) only samples were included. E) Expression peaking in early tadpoles and D5 was only found for the divergent H2B.4 variant. F) Expression of linker histones H1.4 and H1.3 began 1 h pf, peaked at metamorphosis and decreased to D5. G) Genes for the divergent histone variants H2A.3 and H2B.3 are not clustered but were both predominantly expressed in D3/D4 animals. H) Expression of the gene encoding the divergent variant H2A.4.

**Figure 3:**
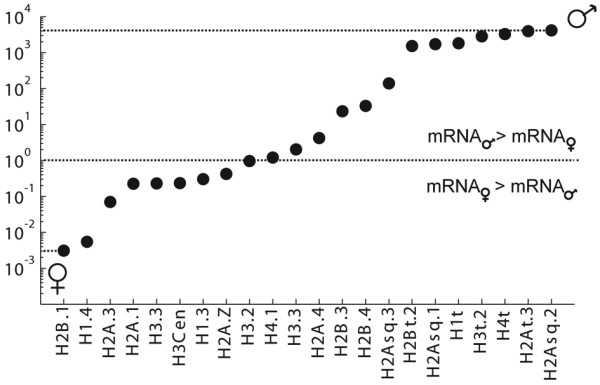
**Transcript levels of selected histone variants in female versus male gonads**. qRT-PCR data is shown as the ratio of testes to ovary transcript levels (y-axis) on a logarithmic scale. Several histone variant transcripts were enriched >5000 fold in testes: H2Bt.2, H2Asq. 1, H1t, H3t.2, H4t, H2At.3, and H2Asq.2.

**Figure 4:**
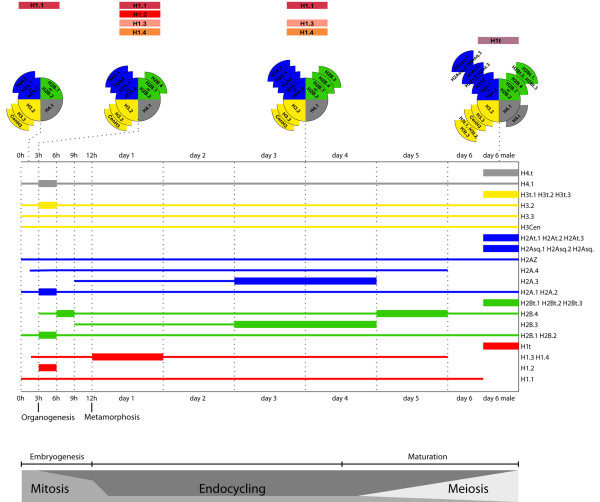
**Histone deployment throughout *O. dioica *development**. Developmental times are indicated with reference to predominance of different cell cycle types (bottom). Cartoons (top) illustrate potential combinatorial possibilities of histone core (wedges: H4s grey, H3s yellow, H2As blue, H2Bs green), and linker H1 (rectangles: orange to red) variants through development. Histone transcripts are represented as lines with thickened portions indicating significant increases either through greater transcriptional activity of a given gene or contributions from additional genes encoding the same variant. The latter was observed for canonical histones of all types during organogenesis with activation of additional genes. All core and linker histones are represented by at least one male-specific isoform (at right).

**Figure 5:**
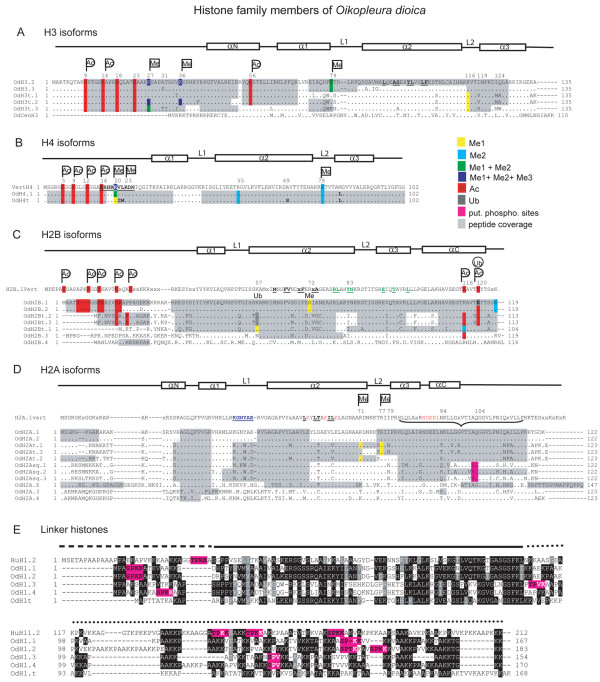
**Protein sequence alignment of *O. dioica *histone variants, including identified PTMs**. Dots indicate identical residues to the top reference sequence for each group. Histone secondary structure motifs are indicated above the reference sequences: loops (Lx), N- and C-terminal tails of the core histones (lines), alpha helices (rectangles), N-terminal tail of H1 (dashed line), H1 globular domain (black line), H1 C-terminal region (dotted line). For H2B and H2A, a vertebrate consensus sequence was generated by aligning canonical histones from chicken, zebrafish, Xenopus and human: "x" indicates variable residues in the consensus. PTMs identified by mass-spectrometry are shaded in different colors as indicated in the key: Me1-3, mono- to tri-methylation, Ac, acetylation; Ub, ubiquitinylation; put. phospho. sites, putative phosphorylation sites. PTMs previously reported from vertebrates are indicated in the consensus sequences. Novel *O. dioica *modifications are shaded without a corresponding PTM-tag in the reference sequence. Some intra- and inter-nucleosomal interaction sites are indicated in different colored bold with underlines: A) H3 α2 interaction (black) with α2 of H4. B) H4 residues 16-25 (black) interact with the acidic patch of H2A. C) H2B residues (black) interacting with the α2 of H2A and H2B residues (green) that interact as part of a 4-helix bundle with H4. D) H2A residues within L1 that form the H2A self-dimerization domain (blue), residues interacting with the α2 of H2B (black), docking domain that makes contact with the H3-H4 tetramer (bracket) and residues in the acidic patch on the nucleosome disc surface (orange).

**Figure 6:**
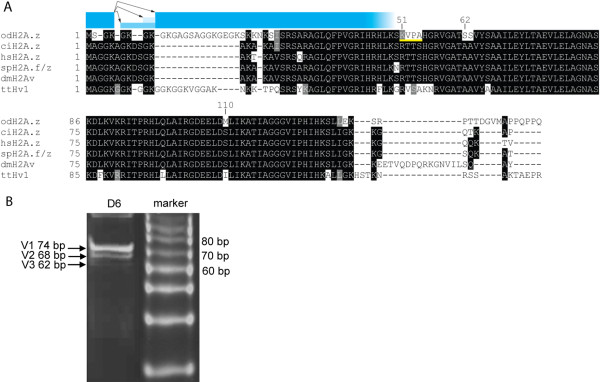
***O. dioica *expresses three H2A.Z N-terminal tail splice variants**. A) A common first exon donor site can be alternatively spliced (arrows) to three different acceptor sites in the second exon of OdH2A.Z "GK" splice variant amino acid sequences, resulting in 3 different ORFs encoding 3 H2A.Z sequence variants. Variants, V1 (blue), V2 (light blue) and V3 (dark blue) contained 4, 3 and 2 N-terminal GK motifs, respectively. Compared to H2A.Zs of other species (ci, *Ciona intestinalis*; hs, *Homo sapiens*; sp, *Strongylocentrotus purpuratus*; dm, *Drosophila melanogaster*; tt, *Tetrahymena thermophila*), Od and tt H2A.Zs have extended N- and C-terminal tails. OdH2A.Z-specific substitutions in the self-dimerization domain (underlined in yellow, K51-A54), in the α2 helix (S62-S63) and adjacent to the acidic patch (M110) are indicated. B) A 15% acrylamide gel showing the PCR products for the three splice variants, which were excised, cloned and sequenced for confirmation.

We assessed expression profiles of all histone genes throughout development by qRT-PCR, including maturing males and females. *O. dioica *histone genes were co-regulated in clusters and most genes belonging to the same locus showed the same developmental expression profiles (Figure 2). Histone clusters could be assigned to seven expression patterns: 1) throughout the life cycle (Figure 2A) 2) exclusively during organogenesis (Figure 2B), 3) male-specific expression in mature D6 animals (Figure 2C), 4) expression peaking in early tadpoles and male D5 animals (Figure 2E), 5) expression peaking at metamorphosis (Figure 2F), 6) expression primarily in D3 and D4 animals (Figure 2G) and 7) transcripts predominantly present from 1 h post-fertilization (pf) to D4 (Figure 2H). Histone isoforms exhibiting multiple changes in amino acid sequence were commonly male-specific. However, some divergent core histone variants displayed expression profiles that preceded gonad maturation in D3/D4 adults (H2A.3 and H2B.3) (Figure 2G), from 1 h pf to D4 (H2A.4) (Figure 2H) or after organogenesis and in late adults at D5 (H2B.4) (Figure 2E).

To improve resolution of germline-specific expression of different histone proteins, maturing D5/D6 animals were dissected and cDNAs prepared separately from testes, ovaries and trunks. Expression ratios were up to 4300-fold higher in testes and 330-fold higher in ovaries for certain isoforms (Figure 3). Extrapolation of ovary-specific transcripts to oogenic function is complicated by maternal mRNA storage in oocytes during vitellogenesis. Taking this into account, only H2A.3 was clearly significantly enriched in the ovary (Figure 3).

The potential combinatorial possibilities of histone core and linker isoforms based on their transcript levels throughout development are schematically summarized in Figure 4. During organogenesis, transcript levels of all canonical histones were increased by the activation of additional, organogenesis-specific histone gene loci. At least one male specific isoform exists in each of the five histone families, including a male-specific H4t isoform.

### Mass spectrometry of *O. dioica *core histones from mature sperm and whole animals

*O. dioica *core histones were prepared for mass spectrometry from D4 and D6 animals, which include endocycling nuclei of the epithelium, the mitotic nuclei of D4 male gonads and female and male meiotic nuclei of D6 animals (Additional File 1 Fig. S2). Histone sequence coverage by LC-MS/MS ranged from 51% (H3) to 90% (H4) and most peptides were identified several times (Figure 5). Many conserved PTMs associated with transcriptional activity and silent chromatin in other species were identified. The N- and to a lesser extent, C-termini, of *O. dioica *H3, H4 and H2B contained numerous acetylated lysine residues (Figure 5 and Additional File 1, Fig. S3). Conserved sites for mono-, di-, and trimethylations such as H4K20, H3K27, H3K36, H3K79 and ubiquitylation of H2BK114 (H2B.1K120 in mammals) were observed, though trimethylation of H4K20 and H3K79 were not detected. Peptides were found for canonical H2B.1 and/or H2B.2 monomethylated at R72 and dimethylated at their last C-terminal K residues as well as the dimethylation of H4 at R55. We found few or no peptides unique to the divergent variants H2B.3, H2B.4, H2A.3, H2A.4 and CenH3, possibly due to low abundance. The large number of histone variants expressed in testes led us to investigate their retention in mature sperm. Mass spectrometry of histones isolated from mature sperm revealed many peptides specific for canonical and male-specific *O. dioica *histone isoforms (Additional File 1, Table S2).

### *O. dioica *core H4s

Whereas other multicellular organisms express a single H4 isoform, *O. dioica *expresses two histone H4s, canonical H4.1 and a male-specific H4t. The major H4.1 subtype was expressed from five genes, with three expressed throughout development and genes at loci II and XII peaking at organogenesis (Figure 1). The H4t gene, located in a male-specific histone gene cluster (Figure 1 and 2C) was specifically expressed in D6 males, with a >3200 enrichment in testes compared to ovaries (Figure 3). H4t-specific peptides were found by mass spectrometry in *O. dioica *sperm histones (Additional File 1, Table S2). Within the H4t amino acid sequence V21 and L22 are replaced by I21 and M22, in a region of the H4 N-terminal tail that interacts with the acidic patch of the adjacent H2A-H2B dimer (Figure 5B). There is also a third residue-change within the α2 helix, where A69 is replaced by S. Based on H4 structure, S69 is not solvent accessible, facing towards the alpha 3 helix of H4.

### *O. dioica *H2B isoforms are expressed before onset and during spermatogenesis

No replacement variants have been identified among H2Bs and all OdH2B genes are intron-less and contain the SL in their 3'UTR (Additional File 1, Table S1). OdH2B.1 and H2B.2, were expressed throughout development (Figure 1), while the other 5 H2B isoforms showed different levels of enrichment in testes. Whereas H2Bt.1, H2Bt.2 and H2Bt.3 were exclusively expressed in testes, with an increase up to >3500-fold compared to ovaries (Figure 3), the divergent H2B.3 gene was expressed at D3/D4, preceding spermatogenesis (Figure 2G) and divergent H2B.4 expression peaked in early tadpoles and D5 (Figure 2E). Intriguingly, these two divergent H2B isoforms display complementary expression patterns (Figure 2E vs 2G) centered around the D3/D4 transition when germline differentiation commences. H2B.3 and H2B.4 have reduced sequence identity to somatic *O. dioica *H2B.1 (65% and 40%, respectively) but do not show significant similarities to testes-specific H2BFWT of mammals, which also shows reduced sequence identity (45%) to mammalian somatic H2B [28]. Mammalian testes-specific H2Bs of the type TSH2B/TH2B have few residue changes compared to their canonical counterparts, but all share three substitutions within their HFD, I41, G60 and N67. Interestingly, canonical OdH2Bs share these same residue substitutions and therefore have slightly higher sequence similarity to testes-specific rather than somatic H2Bs of mammals (Additional File 1, Fig. S4). Structurally, all OdH2B proteins (Figure 5C) have short N-terminal tails compared to other chordate H2Bs and none of the isoforms contain K/R-rich motifs characteristic of H2Bs retained in echinoderm sperm [29].

### *O. dioica *core H3s

The *O. dioica *H3 complement comprises all universal H3 variants but lacks mammalian-specific H3.1. OdH3.2 and H3.3 are 100% identical in amino acid sequence to vertebrate orthologs (Figure 5A). Additionally, *O. dioica *expressed three male-specific H3 isoforms (H3t.1, H3t.2 and H3t.3) highly enriched in testes (Figure 3). H3t.1 and H3t.2 contained a fully conserved SL and were co-regulated within a male-specific histone gene cluster (Figure 2C, 1 locus XVII and IX), whereas H3t.3 was expressed from an orphan gene with a degenerate SL (Additional File 1, Table S1), indicative of a replacement variant.

Within chordates, testes-specific H3 variants have only been reported from mammals. OdH3ts, however, are more divergent than mammalian H3ts when compared to canonical H3.1/H3.2. OdH3t.3 has three residue substitutions in the N-terminal tail and ten in the histone fold, whereas mammalian H3ts exhibit only one and two substitutions, respectively. OdH3ts and mammalian H3ts share no common residue changes and phylogenetic analyses indicate that the H3ts of *O. dioica *are more closely related to other OdH3s than to mammalian H3ts (data not shown). We did not find similar H3t genes within the genomes of the two *Ciona *species, suggesting these isoforms might be specific to the appendicularian lineage. A notable substitution within the N-terminus of H3t.3 is the change of A31 to a phosphorylatable T residue. The same position in H3.3 is S, which is phosphorylated in mitosis and meiosis and is the only known H3.3-specific modification [30,31]. Within the histone fold, the most apparent sequence changes of the OdH3ts are exposed on the disc surface of the nucleosome, surrounding K79, a conserved site for methylation which was present in *O. dioica *canonical H3s (Figure 5A). Further residue changes occur at positions relevant for intranucelosomal interactions. Within loop 2 (*L2*) and α-helix 3, I119 and I124, which make contact with loop 1 (*L1*) and α-helix 3 of H4, respectively, are replaced by V119 and E124.

Another intron-containing H3 gene lacking the SL encodes a divergent H3 replacement variant that we identified as the *O. dioica *centromeric H3. OdCenH3 shows characteristic CenH3 features: a very divergent N-terminal tail, its *L1 *loop is extended and F84 in *L2*, which interacts with the *L1 *of H4, is replaced by W. When expressed as an eGFP fusion protein, OdCenH3 specifically localized to the centromeres of *O. dioica *chromosomes [32].

### *O. dioica *H2As: H2A.Z-splice variants but no H2AX

Similar to vertebrates, the H2A family is the largest and most diverse group of core histones in *O. dioica*. H2A.1, encoded by 5 genes, exhibits the highest identity to canonical vertebrate H2As. H2A.1 and H2A.2, both expressed throughout development, differ by a single amino acid change from A to S at position 13 (confirmed by ESTs). *O. dioica *also expresses six male-specific isoforms (H2At.1, H2At.2, H2At.3, H2Asq.1, H2Asq.2 and H2Asq.3) and two divergent replacement variants, H2A.3 and H2A.4, lacking the SL (Additional File 1, Table S1). Comparing the H2Asq and H2At proteins with each other and with H2A.1, they mainly differ in their N-terminal tails and their docking domain, which interacts with the H3-H4 tetramer (Figure 5D). Nevertheless, all male-specific H2As share residue changes in structurally important regions such as: the H2A self-dimerization domain, within *L2 *that interacts with the H2B *L1*, DNA contacts, and residues in the α2 helices that interact within the H2A-H2B dimer. OdH2As have short C-terminal tails compared to vertebrate H2As.

No macroH2A gene was identified in *O. dioica*. More surprising was the finding that no OdH2As contained the PIKK substrate SQE/DΦ phosphorylation motif that characterizes all H2AXs. Three male-specific *O. dioica *H2A isoforms (H2Asq.1, H2Asq.2, H2Asq.3) contain a putative phosphorylation SQ-motif, adjacent to the C-terminal alpha helix, as a result of a residue change from A104 to S (Figure 5D). Although this SQ-motif could present an alternative phosphorylation site for PIKKs, its location in the docking domain makes it less solvent accessible than the typical position of the SQE/DΦ-motif at the end of the C-terminal tail. We conclude there is no H2AX homolog in *O. dioica*.

The OdH2A.Z variant exhibits the specific divergent characteristics with respect to H2A.1 described for universal H2A.Zs, including a highly positive N-terminal tail, a divergent *L2*, and divergent self-dimerization and docking domains. Further, H2A.Z-specific substitutions are present, including the replacement of N94 with D, which extends the acidic patch, a substitution of E104 by G and the presence of two solvent accessible histidines (H123, H125 in OdH2A.Z). OdH2A.Z differs from consensus H2A.Z sequences at several residues that are invariant in higher eukaryotes and similar in position to residue changes in *Tetrahymena *H2A.Z (Figure 6A). These include three non-conservative amino acid substitutions in the self-dimerization domain (K50-A53 in OdH2A.Z), two S-residues (S61, S62) replacing two A-residues within the α2 helix and substitution of S by M at position 109, adjacent to the acidic patch. Similar to *Tetrahymena *H2A.Z, the OdH2A.Z C-terminal tail lacks one of two conserved K-residues that are mono-ubiquitylated in mammalian heterochromatin. Another atypical feature of OdH2A.Z is extension of a comparatively short H2A.Z C-terminal tail by 10 amino acids containing a "PPQPPQ" motif.

A further similarity to *Tetrahymena *H2A.Z is the long N-terminal tail of OdH2A.Z, extended by 11, mainly G, K, and A residues. H2A.Z N-terminal tails of higher eukaryotes harbor several GK-repeats that can be acetylated (Figure 6A). Intriguingly, we found that *O. dioica *is able to modify the number of GK-motifs within its H2A.Z N-terminal tail by alternative splicing. Alternative splice acceptor sites within the second exon of the OdH2A.Z gene, result in shortening of the N-terminus by precisely one (H2A.Zb) or two (H2A.Zc) of the first three GK-pairs (Figure 6B). Full length OdH2A.Za was predominantly expressed, but all three isoforms were present throughout development.

We also identified two additional H2A variants that did not resemble any universal or lineage-specific H2A variants. H2A.3 and H2A.4 showed 78% amino acid sequence identity to each other but ≤55% identity with other OdH2As. From the N-terminal tail to the α2 helix, H2A.4 and H2A.3 show almost no amino acid sequence conservation to canonical H2A.1. Similar to H2A.Z, both variants differed significantly in the self-dimerization domain, within *L2 *and in their docking domains, but do not show typical features in primary structure described for the mammalian H2A.Bbd. As for the OdH2B subtype H2B.3, H2A.3 was predominantly restricted to D3/D4 animals (Figure 2G) and transcripts were enriched in the ovary (Figure 3). Expression of H2A.4 commenced after zygotic gene activation and decreased significantly when animals began to mature after D4 (Figure 2H).

### *O. dioica *linker H1s

We identified 5 OdH1 genes encoding 5 different H1 proteins. With the exception of D6 males, H1.1 was expressed at every stage of development (Figure 4), while H1.3 and H1.4 were expressed from 1 h pf to D5, peaking during metamorphosis (Figure 2F). H1.1, H1.3 and H1.4 were the dominant H1s throughout development (Figure 4), while H1.2 and H1t were restricted to organogenesis and testes, respectively (Figure 2A,C). The H1.1, H1.2 and H1t genes are intron-less, located within histone clusters, and contain the SL (Additional File 1, Table S1). Variants H1.3 and H1.4 were expressed from orphan genes that contain one intron and a degenerate SL, suggesting replacement variant function.

Increased content of positively charged residues and tail length have been reported to strengthen binding affinity, whereas phosphorylation at S/TPXK motifs weakens H1 binding and destabilizes chromatin structures [33]. The N-terminal tails of *O. dioica *H1s are short in comparison to H1 subtypes of mammals with H1.1, H1.3 and H1.4 also having short C-terminal tails (Figure 5E). OdH1.2 contains the most extended C-terminus and has the highest content of positively charged residues (31% vs 28% for H1.1, H1.4, H1t and 26% in H1.3). It is also the only *O. dioica *linker histone with two phosphorylation motifs within the C-terminus, while H1.1, H1.3, H1.4 have one. This may suggest that among *O. dioica *linker histones, H1.2, restricted to early development, has the widest range of binding affinities, a feature that may facilitate chromatin remodeling events during embryogenesis. In contrast, OdH1t, the shortest linker histone, lacks the conserved phosphorylation motif, suggesting a more restricted binding affinity range.

## Discussion

Analyses of histone complements of higher eukaryotes have been published for metazoans such as *Drosophila *[34,35], human and mouse [36], chicken [37], *C. elegans *[38]*Xenopus *[39] and sea urchin [40]. Comprehensive developmental expression data is only available for the sea urchin histone complement. Here we show that the genome of the urochordate *O. dioica *encodes a high diversity of histone isoforms. As in other organisms, the canonical histone genes are organized as clusters and share divergent promoters, though in contrast to *Drosophila*, sea urchin and mammals, the histone genes have not remained physically linked in one or two large clusters or tandem repeats. Instead they are dispersed in small groups throughout the genome, similar to the *C. elegans *histone complement. Organization of the *O. dioica *histone complement exhibited limited affinity with the sister urochordate ascidian species, *C. intestinalis *or *C. savignyi*, which in turn, differed significantly from each other. Therefore, developmental mode (e.g. rapid early cleavages), genome size, and phylogenetic relationship do not appear to be overriding determinants of histone gene organization.

### Histone modification patterns and genome size

A current view is that histone modifications become more complex from unicellular eukaryotes to mammals [41,42]. Lysine acetylation of H3 and H4 N-terminal tails, as well as methylated H3K4, H3K36 and H3K79 are considered active marks, while methylated H3K9, H3K27 and H4K20 mark silent chromatin. Combined with previous studies [43,44] the mass spectrometry data now indicate that all conserved "ON" and "OFF" marks are present on *O. dioica *histones. Whereas unicellular eukaryotes, such as yeast, *Tetrahymena *and *Plasmodium *principally exhibit modifications associated with transcriptional activation, mammals also employ a wider range of marks involved in gene silencing [41,42]. The observation that hallmarks of silenced chromatin such as H3K9 and H3K27 methylation are low in yeast and *Tetrahymena *[41] has been explained by the fact that the majority of the genome in unicellular eukaryotes is transcriptionally competent, whereas more than 60% of the mammalian genome is permanently silenced and only ~3% of its DNA encodes structural genes [45]. Considering the high gene density of the *O. dioica *genome (one gene per 4-5 kb) and the short intergenic regions, we also expect the proportion of the *O. dioica *genome that is permanently silenced to be relatively low compared to that of human. However, our analysis revealed no obvious lack of silent heterochromatic marks in *O. dioica*, since methylation of H3K27 (Additional File 1, Fig. S3) and H4K20 were detected by mass spectrometry and methylation of H3K9 has been confirmed by immunofluorescence [44]. In the unicellular eukaryotes, *Plasmodium*, *Tetrahymena *and *S. cerevisiae*, protein coding sequence accounts for ~60, 40, and 70%, of the respective genomic sequences [41,42] whereas they account for ~44% of the genome in the multicellular *Neurospora crassa *[46] and *O. dioica *[24]. Despite a higher proportion of protein coding sequence than *Tetrahymena*, the latter two organisms do not exhibit the same reduction in repressive chromatin marks as the former three, suggesting that augmentation of repressive marks is more associated with increased developmental complexity/cell type specification than with the global percentage of transcriptionally inactive genomic regions per se.

### A developmental cell cycle switch in linker H1 variants

The number of linker histone subtypes identified across multicellular organisms is quite variable compared to core histone families. At least 11 linker histones are expressed in human and mice, the *C. elegans *genome encodes 8 H1 isoforms, but only one linker histone is known in *Drosophila *[22,35]. The 5 OdH1 proteins contain comparatively short N- and C-terminal tails. The only linker histone type with similarly short C- and N-termini in vertebrates is H1.0, a replacement variant present mainly in terminally differentiated cells but also found in cells that have entered a prolonged inter-mitotic period [47]. OdH1.1 and the replacement variants OdH1.3 and OdH1.4 have the shortest C-termini and we found them to be the only H1 isoforms present in the endocycling stages of *O. dioica*, when the majority of cell fates are definitively determined and these cells no longer undergo mitosis. Furthermore, they contain only one S/TPXK motif, while OdH1.2, which is restricted to the mitotic stages of *O. dioica*, has two. Endocycling chromatin may not require the same extent of dynamic modulation as is necessary to regulate a mitotic cell cycle. This may result in the deployment of linker histones with a less dynamic range of binding affinities expected from the short OdH1.1, OdH1.3 and OdH1.4 proteins.

### Absence and innovation in the *O. dioica *histone H2A complement

The absence of H2AX raises interesting questions about genome integrity and the double-strand break (DSB)-repair pathway in *O. dioica*. Rapid phosphorylation of H2AX generating γH2AX [48] is central to both of the alternative DNA repair pathways, nonhomologous end-joining (NHEJ) and homologous recombination (HR), which compete for DSB in eukaryotic cells [49]. Nevertheless, H2AX is dispensable for HR during meiosis in H2AX-deficient mice [50] and the MRE11-Rad50-NBS1 repair complex is able to recruit repair and signaling proteins to DSB sites in the absence of H2AX [51]. HR efficiency is influenced by template accessibility, causing an up regulation of HR during S and G2 phases of the cell cycle when sister chromatids are available [52]. The challenge of locating a homologous template for HR repair may not be an obstacle in the compact genome of *O. dioica*, where up to several hundred copies of each locus [25] are available for recombination in endocycling cells. It is therefore possible that DSBs arising in endocycling cells of *O. dioica *that do not traverse mitosis, could be repaired by an H2AX-independent HR pathway. In support of this, the *O. dioica *genome lacks many key components of the NHEJ pathway [24], including DNA-PK, a key protein that facilitates alignment of non-complementary ends and regulates end-processing. The observation that both H2AX and DNA-PK are present in the sister class ascidian *C. intestinalis*, indicates secondary loss of H2AX in the appendicularian lineage as opposed to urochordates as a whole having failed to evolve an H2AX variant.

H2A.Z is one of the best studied universal core histone variants and, with the exception of CenH3, the only variant found to be essential in several species [18-20]. H2A.Z can be found at active gene loci and the acetylation mark is erased at the onset of mitosis [53], suggesting that acetylation of its N-terminus may be required to lower affinity for DNA and facilitate access for essential competing factors at promoters or to prevent higher order folding. Studies on H2A.Z (hv1) acetylation in *Tetrahymena *revealed that acetylation of the N-terminus works to modulate an essential charge patch [54] and viable transformants could be obtained when all of the six K residues within the N-terminus of H2A.Z were mutated to R as long as a charge reducing mutation was also included. This suggests that the function of H2A.Z acetylation in *Tetrahymena*, in contrast to a site-specific histone code, is to alter the charge of the N-terminal domain. It is therefore intriguing that *O. dioica *uses alternative splicing to control the number of acetylatable K residues in the N-terminus of H2A.Z. Deployment of H2A.Z splice variants with altered numbers of acetylatable K residues would seem less dynamic than simply modulating the acetylation status of a fixed number of K-residues. However, in the endoreduplicative cell cycle variant used predominantly for growth in *O. dioica*, genes are disrupted transiently by passage of the replication fork but do not undergo the extensive condensation cycles occurring during the proliferative mitotic cell cycles characteristic of growth in most chordates. On the other hand, a challenge during non-polytene endocycles is to coordinately regulate hundreds of copies of a given gene dispersed in a single interphase nucleus [55]. In this situation, more fixed stepped levels of charge, with a reduced dynamic modulation range may be advantageous. The existence of two non-redundant H2A.Z variants in vertebrates [56] displaying subtle differences in their association with post translationally modified canonical histones and chromatin localization [57] suggests that generation of different H2A.Z variants might not be an uncommon strategy in animals to further extend the functional repertoire of H2A.Z proteins. As several metazoan H2A.Zs contain a conserved intron directly following the start ATG and possess multiple alternative splice acceptors within their second exon, it will be of interest to determine whether generation of alternative splice H2A.Z variants is not unique to *O. dioica*.

### Variant male nucleosomes in *O. dioica*

*O. dioica *has a particularly high number of histone isoforms expressed exclusively or highly enriched in testes, including 6 H2At, 5 H2Bt, 3 H3t and 1 H4t. Testes-specific histones are poorly studied but are thought to substitute for canonical histones in meiotic and post-meiotic cells prior to replacement of histones by protamines [58]. Analyses of mammalian testes-specific H2As, H2Bs [59,60] and H3s [61] indicate that nucleosomes containing these variants are less stable than those composed of canonical histones. *O. dioica *histone isoforms exclusively expressed in testes (H2At.1-H2At.2, H2Asq.1-H2Asq.3, H2Bt.1-H2Bt.3, H3t.1-H3t.3) might affect intranucleosomal interactions. *O. dioica *H2At and H2Bt histones exhibit alterations in regions that promote stabilization of interactions with the H3-H4 tetramer and within the H2A-H2B dimer. Furthermore, several of the OdH2Bts have shortened N-terminal tails, lacking the first 14-21 N-terminal residues (H2Bt.2, H2Bt.3), predicted to form an α-helix in vertebrate H2Bs (residues 10-21) [62]. The N-terminal tails of mammalian H2Bs are involved in interactions with DNA, internucleosomal histone-DNA interactions [63] and are important for the mitotic and apoptotic condensation of chromosomes. Very divergent or short tails may be compromised in some of these functions and result in further loss of nucleosome stability. These features might provide the basis for structural transitions and assembly of genomic sub-regions into altered DNA-packaging structures and/or facilitate histone mobilization during male pronuclear reprogramming.

### Is there a testes-specific histone code in *O. dioica*?

The massive synthesis of histone variants, including testes-specific members, and the stage-specific posttranslational modification of histones during spermiogenesis have led to proposal of a testes-specific "histone code" generated by combining both histone variants and PTMs [64]. In zebrafish sperm, genes with embryonic functions are embedded in distinctive, complex and atypical chromatin structures [65]. Hyperacetylation of histones is associated with histone replacement in early elongating spermatids [66] and has been reported for some testes-specific variants such as rat TH2B [67]. Consistent with this, we found several K residues within the N- and C-termini of OdH2Bt.1 and H2Bt.2 to be acetylated. Moreover, *O. dioica *tiling array data confirm expression of a bromodomain-containing testes-specific factor (BRDT) [32] that induces chromatin remodeling in the presence of histone hyperacetylation in mammals [68], possibly mediating nuclear reorganization during spermiogenesis.

Residues exposed on the nucleosome disc surface may in part account for the extreme evolutionary constraints on histone sequences, since they interact with nuclear factors and mediate internucleosomal interactions. Dot1 is the methyltransferase responsible for mono-, di-, and trimethylation of H3K79, a residue exposed on the disc surface within the globular domain of H3. H3K79 methylation (H3K79me) is found in euchromatin [69] but the modification also plays an important role in heterochromatin formation in mice and yeast [70]. Interestingly, the residues surrounding K79 are substituted in all male-specific H3ts of *O. dioica*. In H3t.3, not only have the surrounding residues been modified, but K79 has been replaced by R, raising the possibility that the OdH3t isoforms lack K79 methylation. These residues are nearly invariant across species, though substitutions are present in pollen-specific H3s of *Arabidopsis *and rice, *C. elegans *H3.3 variants, and all H3s identified in *Plasmodium *and *Tetrahymena *(Additional File 1, Fig. S5). The fact that both Dot1 and K79 methylation are absent in *Arabidopsis *[71,72] and *Plasmodium *[42,73] may suggest that Dot1 binding could partially account for evolutionary constraints on these residues. These residues likely create a specialized nucleosome surface for Dot1 binding, as single mutations centered directly around H3K79 distinctly affected the three typical forms of yeast silencing (rDNA, telomere, and silent mating loci silencing; [74]). A single R79 substitution, as in OdH3t.3, weakened silencing of rDNA and the silent mating loci but enhanced silencing at telomeres. Together with the surrounding mutations found in the other OdH3t variants this may indicate an inability of Dot1 to methylate H3K79 in OdH3ts.

No H4 sequence variant has been described in higher metazoans and the fact that *O. dioica *males express a H4 sequence variant appears unique. Expression of a male-specific H4 gene has been reported in rats [75], but this transcript encodes an invariant H4 protein sequence. Two of the residue changes in OdH4t occur within the N-terminal tail adjacent to the H4K20 methylation site, a core histone modification exposed on the nucleosome disc surface. H4K20me^3 ^affects internucleosomal interactions resulting in more compact higher order chromatin structure [76]. In view of the extreme conservation of the H4 amino acid sequence across metazoans, also preserved in the other 5 *O. dioica *H4.1 genes, a neutral function of these mutations is questionable.

## Conclusions

The traditional view of histones as slow evolving proteins has changed considerably within the last decade. It is now clear that histone variants have diversified in many species to assume crucial roles in transcriptional activation, DNA repair, chromosome segregation and other processes. Recent data from more exotic model organisms such as trypanosomes [77] and rotifers [78] increasingly link histone complement evolution to specific life history traits of organisms. The results here show that there is considerable plasticity in histone gene organization and variation within histone families even within the chordate lineage. Of particular note among the high diversity of histone variants in the fast evolving appendicularian lineage, is a concentration of variation in all core and linker histone families associated with differentiation of the male germline, raising intriguing questions regarding the testes as an evolutionary playground for the innovation of histone variants.

## Methods

### In silico- and phylogenetic analyses

BLAST searches of the *Oikopleura *genome [79,80] were conducted using previously identified *Oikopleura *core histone fold domain sequences or linker H1 globular domain sequences [26] and H1 tunicate homologs. Since these searches failed to isolate H2AX and macroH2A variants, additional searches were performed including the H2AX consensus motif SQE/DY and the *Xenopus *macro H2A sequence, respectively. Sequences were confirmed by ESTs, RACE or sequencing from qRT-PCR products as indicated on the *Oikopleura *histone webpage [81]. Methodological details on the phylogenetic analyses of *Oikopleura *histone gene families are given in the legend of Additional File 1, Figure 1. Amino acid substitutions were evaluated using 3D-structure predictions for *Oikopleura *histone variants obtained from I-tasser [82] with the crystal structure of the *Xenopus *nucleosome specified as a template (PDB file *1kx5:A *for histone H3, *1kx5:B *for histone H4, *1kx5:C *for histone H2A and *1kx5:D *for histone H2B). PDB files of *Oikopleura *histone variants were viewed and aligned to PDB files of nucleosome crystal structures (PDB files 1kx5 and 1f66) in Pymol (Version 1.2r3pre, Schrödinger, LLC). Databases and browsers used for analyzing histone genes: Ensembl [83], Histone database [84], *Ciona savignyi *database [85], *Ciona intestinalis *JGI [86] and Ghost [87], and *Strongylocentrotus purpuratus *[40].

### Animal culture and collection

*O. dioica *culture was performed as described [88]. For *in vitro *fertilizations, females were collected in watch glasses, washed with artificial seawater (Red Sea, final salinity 30.4-30.5 g/l) and left to spawn. Sperm from 3-5 males was checked for viability and used for fertilization. Embryos were left to develop at room temperature (RT) and subsequently frozen in liquid nitrogen. D2 - D6 animals were removed from houses and anaesthetized in seawater containing 3-aminobenzoic acid ethyl ester (MS222, Sigma, 0.125mg/ml). For qRT-PCR at all developmental stages, animals from the same population were used. Two replicate populations were characterized. For preparation of histones, 4000 D4 and 1000 D6 animals were poured against a glass plate to separate them from their houses and collected in 1 L glass beakers. Sea water was replaced two times by 500 ml of seawater-MS222 and beakers left on ice for 10 minutes to sediment and harvest the animals. Mature sperm was collected by allowing 200 males to spawn in Petri-dishes and the sperm-sea water suspension was collected in 5 ml falcon tubes. Sperm was sedimented in a Sorvall centrifuge (RT 6000 D), 3000 rpm at 4°C for 10 min. For testes and ovary-specific samples, animals were selected and dissected as in [89].

### Quantitative reverse transcriptase-polymerase chain reaction (qRT-PCR)

RNA extraction and first strand cDNA synthesis was performed as previously [90]. cDNAs from the two populations were controlled for genomic contamination including RT- controls. Ribosomal protein 23 and elongation factor 1-beta expression levels were used as normalization controls using the comparative method of relative quantification [91]. Histone primer (Additional File 1, Table S3) specificities were confirmed by sequencing directly from qRT-PCR products. After initial denaturation for 15 min at 95°C, 40 cycles of 95°C for 15 sec, 55°C or 58°C for 30 sec and 72°C for 30 sec were conducted, with a final extension for 5 min at 72°C.

### Cloning of H2A.Z-splice variants

PCR was performed with CC212 forward (5' GGCGCGCAACTGAGAGAAATC 3') and CC213 reverse (5'CCAGCACTTCCGGCACC 3') primers, flanking the alternative splice sites with 5 μl D6 cDNA as template. After initial denaturation for 5 min at 95°C, 35 cycles of 95°C for 20 sec, 56°C for 15 sec and 72°C for 15 sec were conducted, with a final extension for 3 min at 72°C. PCR products were run on a 15% acrylamid gel with a 10 bp ladder (Promega). Bands were excised and acrylamid slices crushed in 400 μl TE, 8.2 μl 5M NaCl with subsequent nucleic acid precipitation by adding 1/10 vol 3M NaAc and 2.5 vol cold 100% EtOH. Precipitated PCR fragments were cloned into the Topo vector with the TOPO TA Cloning kit (Invitrogen) according to manufacturer's instructions.

### Preparation of *O. dioica *histones

Histones were extracted using a histone purification kit (Active Motif; #40025) according to manufacturer's instructions and by the acid extraction method. For acid extraction, Lysis buffer [1 × PBS, 0.5% Triton X 100 Sigma, 5 mM PMSF, 100 mM NaCl, 10 mM Sodiumbutyrate, 1× Phosphatase inhibitor cocktail (Sigma, P5726) was added to frozen sperm or D4/D6 animals. D4/D6 animals were dounced (Kontes Glass Company, 749520-0000) in 1.5 ml eppendorf tubes and sperm was sonicated in the lysis buffer (vibra cell, amplitude 40, 2 mm sonicator). HCl was added to 0.2 N and tubes were shaken vigorously for 2 h (D4/D6) at room temperature or overnight (sperm) at 4°C. Insoluble material was removed by centrifugation at 13000 rpm at 4°C for 15 min and Trichloracetic acid was added to 25% (vol/vol) to precipitate histones for 30 min at 4°C. Precipitates were collected by centrifugation at 14000 rpm for 15 min at 4°C and the supernatant was saved for later analysis. Histone pellets were washed twice with 500 μl acetone containing 0.1% HCl and dissolved in Laemmli loading buffer for subsequent mass-spectromery and silver gel analyses. Purified histones were loaded on 15% SDS-PAGE gels and stained with the Silver Stain Plus kit (Bio-Rad # 161-0461).

### Mass spectrometry

Histones were loaded on 15% SDS-PAGE gels, excised, reduced and alkylated [92]. Digests were performed overnight with Trypsin (Promega) following elution with trifluoroacetic acid. Peptides were analysed using a nano-high-pressure liquid chromatography Aligent 1100 nanoflow system connected online to a 7-Tesla linear quadruple ion-trap Fourier transform mass spectrometer (ThermoElectron) [92]. Peptides were identified using the Mascot algorithm (MatrixScience) to search a database containing the histone sequences of *Oikopleura*. Initial search criteria were: Trypsin as enzyme; peptide charge 1+, 2+, and 3+ with a peptide tolerance of 10 parts per million for the parental peptide and 0.6 Da for fragmentation spectra. Fixed modifications were set to fixed carbamidomethyl modification for cysteines. Oxidation of methionine, deamidation (Q and N), acetylation (K), mono- and dimethylation (K and R), trimethylation (K), phosphorylation (S, T and Y), and ubiquitinylation (K) were searched as variable modifications. Parent ion masses were internally calibrated by MSQuant [93] to obtain accuracy better than five parts per million.

## Competing interests

The authors declare that they have no competing interests.

## Authors' contributions

AM, EMT and CC identified the histone complement, AM and MR performed the developmental expression profiles, PWTCJ and HGS performed the mass spectrometry, CC and CN assessed H2A.Z alternative splicing, AM, CC and EMT designed experiments and analyzed the data, AM and EMT wrote the manuscript. All authors approved the final manuscript.

## Supplementary Material

Additional file 1 **Additional Tables S1-S3 and additional Figures S1-S5**. **Table S1**. *Oikopleura dioica *histone genes: stem-loop sequences and expression profiles. **Table S2**. Histone variants retained in mature *Oikopleura dioica *sperm. **Table S3**. Primer pairs used for quantitative RT-PCR. **Figure S1**. Phylogenetic relationships among *Oikopleura dioica *histone genes. **Figure S2**. Histones extracted from D4 and D6 animals. **Figure S3**. Spectra of *O. dioica *histone modifications obtained by LC-MS/MS. **Figure S4**. Alignment of mammalian somatic and testes-specific H2B variants with the canonical H2B of *O. dioica*. **Figure S5**. Histone H3 isoforms of species with substitutions surrounding the methylation site at K79. **Additional Data References**. Click here for file
