# Supplementary material for: Histone variant innovation in a rapidly evolving chordate lineage
Source: BMC Evol Biol. 2011 Jul 15;11:208. doi: 10.1186/1471-2148-11-208 (PMC3156773; doi:10.1186/1471-2148-11-208)
Supplement: Additional file 1 — Additional Tables S1-S3 and additional Figures S1-S5. Table S1. Oikopleura dioica histone genes: stem-loop sequences and expression profiles. Table S2. Histone variants retained in mature Oikopleura dioica sperm. Table S3. Primer pairs used for quantitative RT-PCR. Figure S1. Phylogenetic relationships among Oikopleura dioica histone genes. Figure S2. Histones extracted from D4 and D6 animals. Figure S3. Spectra of O. dioica histone modifications obtained by LC-MS/MS. Figure S4. Alignment of mammalian somatic and testes-specific H2B variants with the canonical H2B of O. dioica. Figure S5. Histone H3 isoforms of species with substitutions surrounding the methylation site at K79. Additional Data References. [file 1471-2148-11-208-S1.DOC]

Additional File 1

**Table S1.** *Oikopleura dioica* histone genes: stem-loop sequences and expression profiles.

| **Gene locus** | **Introns** | **SL-sequence in 3`UTR** | **Expression** |
| --- | --- | --- | --- |
|  |  |  |  |
| **Histone H4** |  |  |  |
| H4t (I) | - | aaacc**gg**ccc**tt**t**t**t**a**ggg**cc**acaat | male |
| H4.1 (II) | - | AAGCC**GG**CCC**TT**T**T**C**A**GGG**CC**ACAAA | organogenesis |
| H4.1 (III) | - | AAACC**GG**CCC**TT**T**T**T**A**GGG**CC**ACAAA | all stages |
| H4.1 (VII) | - | aaaca**gg**ccc**tt**t**t**t**a**ggg**cc**acaaa | all stages |
| H4.1 (VIII) | - | AACCT**GG**CCC**TT**T**T**C**A**GGG**CC**ACAAA | nd |
| H4.1 (XII) | - | CCACC**GG**CCC**TT**T**T**C**A**AGG**CC**ACAAA | nd |
|  |  |  |  |
| **Histone H3** |  |  |  |
| H3.2 (II) | - | AAACC**GG**CCC**TT**T**T**C**A**GGG**CC**ACAAA | organogenesis |
| H3.2 (III) | - | AAACC**GG**CCC**TT**T**T**T**A**GGG**CC**ACAAA | all stages |
| H3.2 (VII) | - | AAACA**GG**CCC**TT**T**T**T**A**GGG**CC**ACAAA | all stages |
| CenH3 (V) | 2 | AAACCT**G**CCTG**T**T**T**TCATC**CC**CTGCA | all stages |
| H3t.2 (IX) | - | AAACC**GG**CCC**TT**T**T**A**A**GGG**CC**ACAAA | male |
| H3t.1 (XIII) | - | AAACC**GG**CCC**TT**T**T**T**A**GGG**CC**ACAAA | male |
| H3t.3(XVII) | - | ttcaac**g**tcc**tt**t**t**t**a**gga**c**aaatcg | male |
| H3.3 (XIV) | 2 | ATGCCT**G**CCAC**T**T**T**TTTGAGAAACAA | all stages |
| H3.3 (XVIII) | 2 | GTGAACATCAC**T**TCTTAATGCAACAC | all stages |
|  |  |  |  |
| **Histone H2A** |  |  |  |
| H2A.1 (II) | - | AAATC**GG**CCC**TT**T**T**T**A**GGG**CC**ACATA | organogenesis |
| H2A.1 (III) | - | aaacc**gg**ccc**tt**t**t**t**a**ggg**cc**acaaa | all stages |
| H2A.1 (VII) | - | AAACC**GG**CCC**TT**T**T**T**A**GGG**CC**ACAAA | all stages |
| H2A.1 (VIII) | - | AAACC**GG**CCC**TT**T**T**T**A**GGG**CC**ACAAA | organogenesis |
| H2A.1 (XX) | - | aaacc**gg**ccc**tt**t**t**t**a**ggg**cc**acaaa | all stages |
| H2A.2 (XII) | - | aaccc**gg**ccc**tt**t**t**t**a**ggg**cc**acaaa | n.d. |
| H2Asq.1 (IX) | - | AAACC**GG**CCC**TT**T**T**A**A**GGG**CC**ACAAA | male |
| H2Asq.2 (X) | - | aaaca**gg**ccc**tt**t**t**a**a**ggg**cc**acaca | male |
| H2Asq.3(XIII) | - | AAACA**GG**CCC**TT**T**T**A**A**GGG**CC**ACAGT | male |
| H2At.3 (I) | - | AAACA**GG**CCC**TT**T**T**T**A**GGG**CC**ACAAT | male |
| H2At.2 (VI) | - | aaacc**gg**ccc**tt**t**t**a**a**ggg**cc**acata | male |
| H2At.1 (XI) | - | aaacc**gg**ccc**tt**t**t**tgggtgaggggA | male |
| H2A.4 (III) | - | AGAAAA**G**CCC**TT**T**T**T**A**GGG**C**TAAATA | 1h – D4 |
| H2A.3 (IV) | - | aaacaa**g**ccc**tt**t**t**t**a**ggg**c**tacaaa | D3/D4 peak |
| H2A.Z (XVI) | 4 | CAATCTCTCC**TT**T**T**TTTGCTTTTTTT | all stages |
|  |  |  |  |
| **Histone H2B** |  |  |  |
| H2B.1 (II) | - | AAACC**GG**CCC**TT**T**T**T**A**GGG**CC**ACATA | organogenesis |
| H2B.1 (VII) | - | AAACA**GG**CCC**TT**T**T**T**A**GGG**CC**ACAAA | all stages |
| H2B.2 (VIII) | - | AAATC**GG**CCC**TT**T**T**T**A**GGG**CC**ACAAA | organogenesis |
| H2B.2 (XII) | - | AAACC**GG**CCC**TT**T**T**T**A**GGG**CC**ACATT | organogenesis |
| H2B.2 (XX) | - | aaacc**gg**ccc**tt**t**t**t**a**ggg**cc**acata | nd |
| H2Bt.2 (I) | - | AAACC**GG**CCC**TT**T**T**C**A**GGG**CC**ACAAA | male |
| H2Bt.1 (IX) | - | AAACA**GG**CCC**TT**T**T**T**A**GGG**CC**ACAAT | male |
| H2Bt.1 (X) | - | AAACC**GG**CCC**TT**T**T**A**A**GGG**CC**ACATA | male |
| H2Bt.3 (XI) | - | AAACC**GG**CCC**TT**T**T**C**A**GGG**CC**ACAAA | male |
| H2B.3 (III) | - | CTCAA**GG**CCC**TT**T**T**T**A**GGG**CC**AAATC | D3/D4 peak |
| H2B.4 (VI) | - | aaacc**gg**ccc**tt**t**t**a**a**ggg**cc**atata | ETP and D5 peak |
|  |  |  |  |
| **Histone H1** |  |  |  |
| H1.1 (VII) | - | AAACA**GG**CTC**TT**T**T**C**A**GAG**CC**ACAAA | all stages |
| H1.2 (II) | - | TAAAT**GG**CTC**TT**T**T**C**A**GAG**CC**ACAAA | organogenesis |
| H1.3 (XIV) | 1 | AAATCCTCTT**TT**T**T**T**A**AATA**C**GAAAA | metamorphosis |
| H1.4 (XV) | 1 | CGTTC**G**ATCT**TT**T**T**GTAGTTTGAAAA | metamorphosis |
| H1t (I) | - | AAACC**GG**CCC**TT**T**T**T**A**GGG**CC**ACAAA | male |

Conserved nucleotides in stem-loop formation are in bold. High sequence identity between some genes did not allow certain specific expression profiles to be determined (nd).

**Table S2**. Histone variants retained in mature *Oikopleura dioica* sperm.

| **Histone variants in mature sperm** | **Peptides (tandem MS data)** |
| --- | --- |
|  |  |
| **H4 variants** |  |
| H4.1 | KVLRDNIQGITKPAIR (20 – 35)  DAVTYTEHAK (68 -77) |
| H4t | KIMRDNIQGITKPAIR (20 – 35)  DSVTYTEHAKR (68 – 78) |
|  |  |
| **H3 variants** |  |
| All H3s except H3t.3 | YQKSTELLIR (54 – 63) |
| H3t.1 | KSAPATGGIKKPHR (27 – 40)  ELAQQWK (73 – 79) |
| H3t.2 | KSAPTIGGIKKPHR (27 – 40) |
| H3t.3 | DMWLAR (123 – 128)  YRPGTVALR (41 – 49) |
| H3t.1, H3t.2 or H3t.3 | VTVMPK (117 – 122) |
|  |  |
| **H2B variants** |  |
| H2Bt.1, H2Bt.2 or H2Bt.3 | EIQTACR (73 – 79) |
| H2Bt.2 or H2Bt.3 | ESFGIYIYK (23 – 31)  VLKQVHPDQGISTK (32 – 45)  HAVSEGTKAVSKYTASK (97 – 105) |
| H2Bt.3 | KESFGIYIYK (22 – 31) |
|  |  |
| **H2A variants** |  |
| H2A.1 or H2A.2 | HLQLAIRNDEELNK (81 – 94) |

Most variants with high transcript levels in testes were retained in mature sperm.

**Table S3.** Primer pairs used for quantitative RT-PCR.

| **Gene locus** | **Primer names** | **Primer sequence (5`to 3`)** |
| --- | --- | --- |
|  |  |  |
| **H4 Histones** |  |  |
| H4t (I) | H4tF | GTGAAACATGTCTGGTCGAGGA |
|  | H4tR | GGATCGCGGGCTTGGTGATGC |
| H4.1 (III) | H4.1IIIF | GAGCACATAGAACTGTCAAAT |
|  | H4.1IIIR | GTCTCTTCGTAGATCAAACCAG |
| H4.1 (II) | H4.1IIF | AGTAAAACTTGAACAAATCG |
|  | H4.1IIR | GTAGATCAAGCCGGAGATTC |
| H4.1 (VII) | H4.1VIIF | CGCTCGGCTTGAAGAAATC |
|  | H4.1VIIR | GCAAGTCGTCGAATGGCAGG |
|  |  |  |
| **H3 Histones** |  |  |
| H3.2 (II) | H3.2IIF | ATGGCCCGAACCAAGCAAACC |
|  | H3.2IIR | TAAGCACGTTCTCCTCGGATTC |
| H3.2 (III) | H3.2IIIF | GCAGTCTCATTGCTCACTGAA |
|  | H3.2IIIR | GGATGTCCTTAGGCATGATGG |
| H3.2 (VII) | H3.2VIIF | GGCACCTCGATTCTAAACTCA |
|  | H3.2VIIR | GCACGTTCTCCTCGGATTC |
| CenH3 (V) | CenH3F | GCTTCGACCAGGGAATGT |
|  | CenH3R | CGTTCAAGTTCATGCCCGCA |
| H3.3 (XIV) | H3.3XIV | ATGGCCCGAACAAAACAAACG |
|  | H3.3XIV | GCTCGTTCTCCTCGGATTCGT |
| H3.3 (XVIII) | H3.3XVIII | CAGACTGAATAAATTTTGAGG |
|  | H3.3XVIII | GCATGATGGTAACGCGCTTGG |
| H3t.3 (XVII) | H3t.3F | GATCCTCATTCACTCGACATCA |
|  | H3t.3R | CGAAGATCGTTTCGCCACTGCT |
| H3t.2 (IX) | H3t.2F | CTTCAAAACACTGAAAATCACC |
|  | H3t.2R | AGATCAGACTTCCACTGCTG |
| H3t.1 (XIII) | H3t.1F | CGAGCTCGCACAGCAGTGGAA |
|  | H3t.1R | CTTGGCTTCTCCTCGGATACG |
|  |  |  |
| **H2A histones** |  |  |
| H2A.1 (II) | H2A.1IIF | ATTCGGGTCTTTTTGTTATCTC |
|  | H2A.1IIR | CCCAGTCGGTCGAGTCCAC |
| H2A.1 (III) | H2A.1IIIF | GCGATCCGTCCGAGCTGGA |
|  | H2A.1IIIR | CGGCTCCAACTCGCTCTGC |
| H2A.1 (VII) | H2A.1VIIF | CCCAGTCGGTCGAGTCCAC |
|  | H2A.1VIIR | CTGGGCAATGGTGACGCCA |
| H2A.1 (VIII) | H2A.1VIIIF | GTGAAACCAGAACAACAAGTT |
|  | H2A.1VIIIR | TTGTCTCGTGCTGCGTTTC |
| H2A.1 (XX) | H2A.1XXF | GTGAAACCAGAACAACAACTT |
|  | H2A.1XXR | TGCGAAGCATTCGATGGACT |
| H2At.1 (XI) | H2At.1F | gagttggcgctggtgccc |
|  | H2At.1R | CAGGGTTAATGTTGGGAAGG |
| H2At.2 (VI) | H2At.2F | gaccgctacgaagacgaagcag |
|  | H2At.2R | CCATGTAAACAGGAGCACCTGC |
| H2At.3 (I) | H2At.3F | CCGAGTTGGTGCTGGTGCC |
|  | H2At.3R | GATGTCGTGGGTTGATTCGC |
| H2Asq.1 (IX) | H2Asq.1F | CACATTATGCTCGCTATCGG |
|  | H2Asq.1R | GTGTTCTACCCAGCATCCAC |
| H2Asq.2 (X) | H2Asq.2F | CGATGAACAAGAAGGCCG |
|  | H2Asq.2R | AGAGCAGCGTGGATGCTG |
| H2Asq.3 (XIII) | H2Asq.3F | CTGGACGAAAATCGCTGACA |
|  | H2Asq.3R | TCTTCTTTGGCAATAGCGCAG |
| H2A.4 (III) | H2A.4F | GGCCATCTATCCAAACGAA |
|  | H2A.4R | ACTTCTCAGCAGTCTTCTTTGG |
| H2A.3 (IV) | H2A.3F | CAGGAATCCTTAAAACTAGAA |
|  | H2A.3R | GCACGTCAGATCAAGAAAATTG |
| H2A.Z (XVI) | H2A.ZF | GGCGCGCAACTGAGAGAAATCAAA |
|  | H2A.ZR | ATTCGACCAACTGGGAACTGGAGA |
|  |  |  |
| **H2B Histones** |  |  |
| H2B.1 (II) | H2B.1IIF | GCCACTAAAGGTTCCAAGAAAG |
|  | H2B.1IIR | TCGTTGGCGATGCGCTCG |
| H2B.1 (VII) | H2B.1VIIF | GCCACCAAAGGATCGAAGAA |
|  | H2B.1VIIR | GATTCCTTTCGCTTGGCACGTC |
| H2B.2 (VIII) | H2B.2VIIIF | AAGCCATGTCGATCATGAAC |
|  | H2B.2VIIIR | CAAGTTCTCCGGGTAGGCG |
| H2B.2 (XII) | H2B.2XII | ATGGCAGCTACCAAAGGATCA |
|  | H2B.2XII | GAGGAGATACCGGTATCGGGG |
| H2Bt.1 (IX) | H2Bt.1IX | GCAAGTACAACAAAGCACGAA |
|  | H2Bt.1IX | GATCTTGGCAAGGCGGGAAG |
| H2Bt.1 (X) | H2Bt.1X | GCTATGTCCATCATGAACTCGT |
|  | H2Bt.1X | CTCTCGGGAGGAGATAGTGG |
| H2B.t2 (I) | H2B.t2F | GTCAATAACATCGAGAATG |
|  | H2B.t2R | CTTTTCGGTACTTTCGGGCAC |
| H2Bt.3 (XI) | H2Bt.3XI | GCCCGCAAGTACCGAAAATCC |
|  | H2Bt.3XI | CAAAGATGTCGTTGACGAAGG |
| H2B.3 (III) | H2B.3F | GCATCGAGGAAAGGCAAGGA |
|  | H2B.3R | CATGATCGACATCGCATTC |
| H2B.4 (VI) | H2B.4F | GCACGTAAACCGCAGAACCAGT |
|  | H2B.4R | CGGTGGCACGATTTCAAGAGC |
|  |  |  |
| **H1 Histones** |  |  |
| H1t (I) | H1tF | GGCTCCTACCACCGCTACCAAG |
|  | H1tR | CGAAGTTGGCGACAATGTAC |
| H1.1 (VII) | H1.1F | CGATCACCCAACTTACCACGTC |
|  | H1.1R | AGGGCCTTCTTGAGATGGTG |
| H1.4 (XV) | H1.4F | CCAAGTCCGCTGCCAAGG |
|  | H1.4R | GATTTTTCTGCTCAGCGGAGAT |
| H1.3 (XIX) | H1.3F | CCGCGAAGCCCGTTACCAAG |
|  | H1.3R | GTCCTTGTGCTCAGAAAAGATC |
| H1.2 (II) | H1.2F | GCATCGGAGCTTCAGGTTCA |
|  | H1.2R | GACAGGCTTTTTAGTCGCAGTA |

Primer pairs specific for H4.1XII, H4.1VIII, H2A.2XII and H2B.2XX could not be designed due to the high sequence identities between genes.


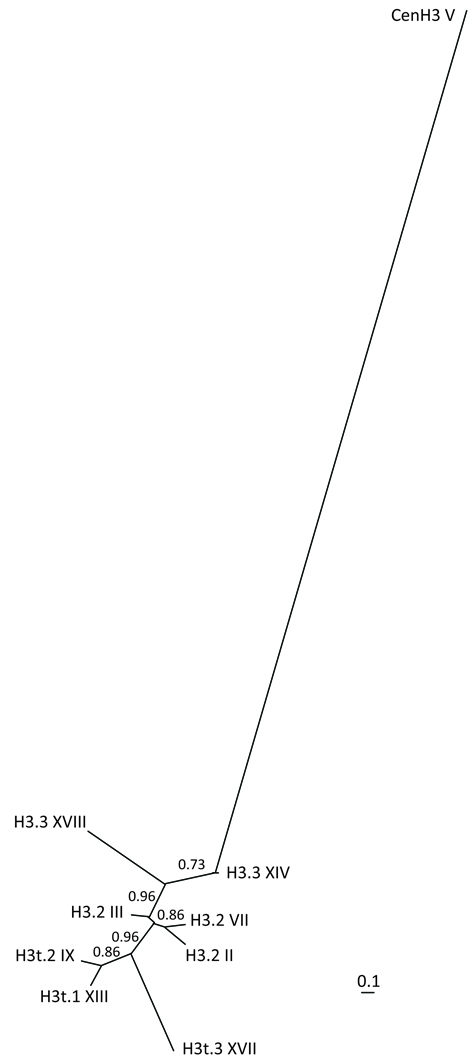

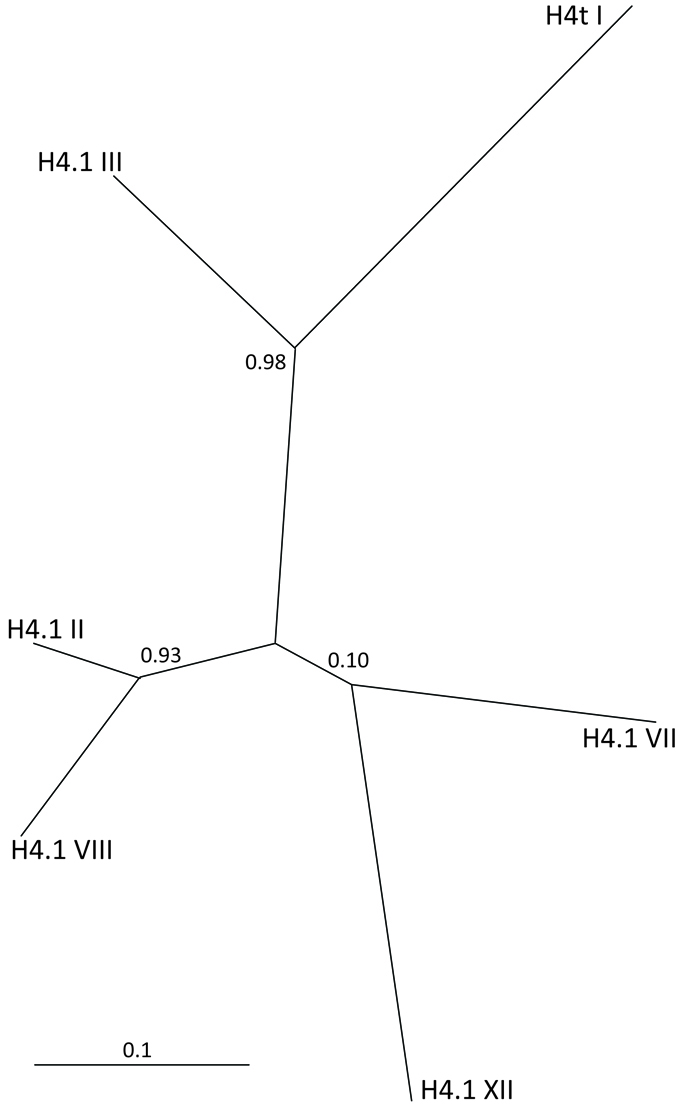

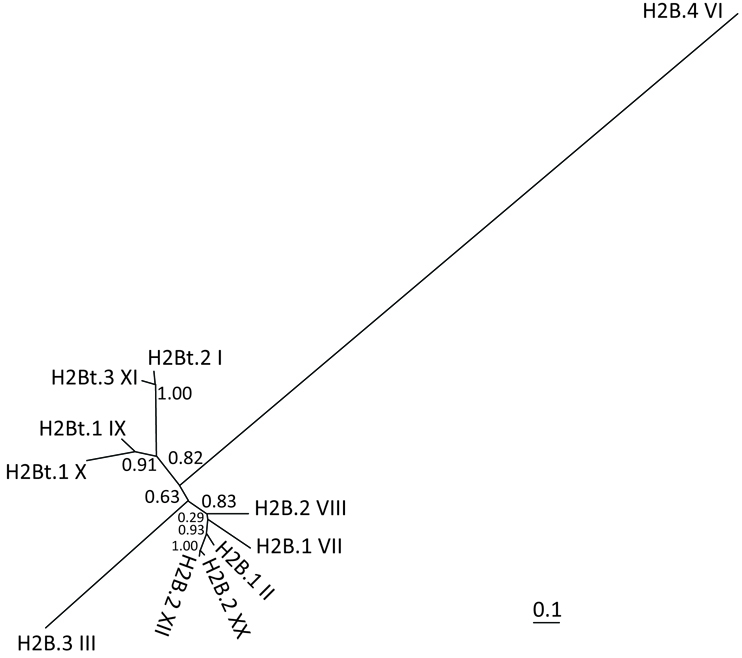

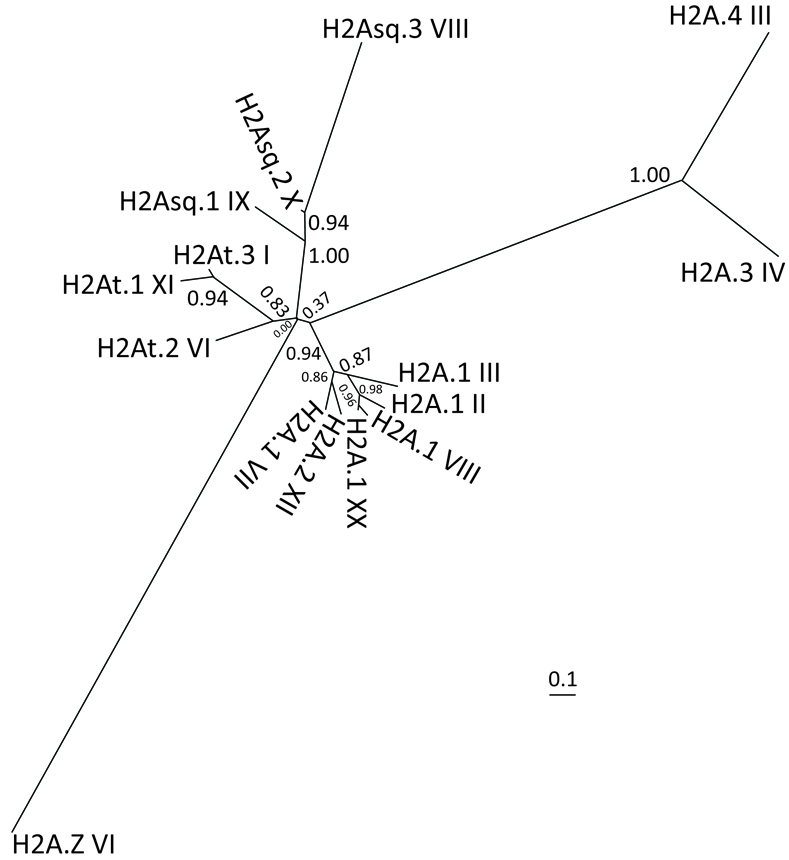

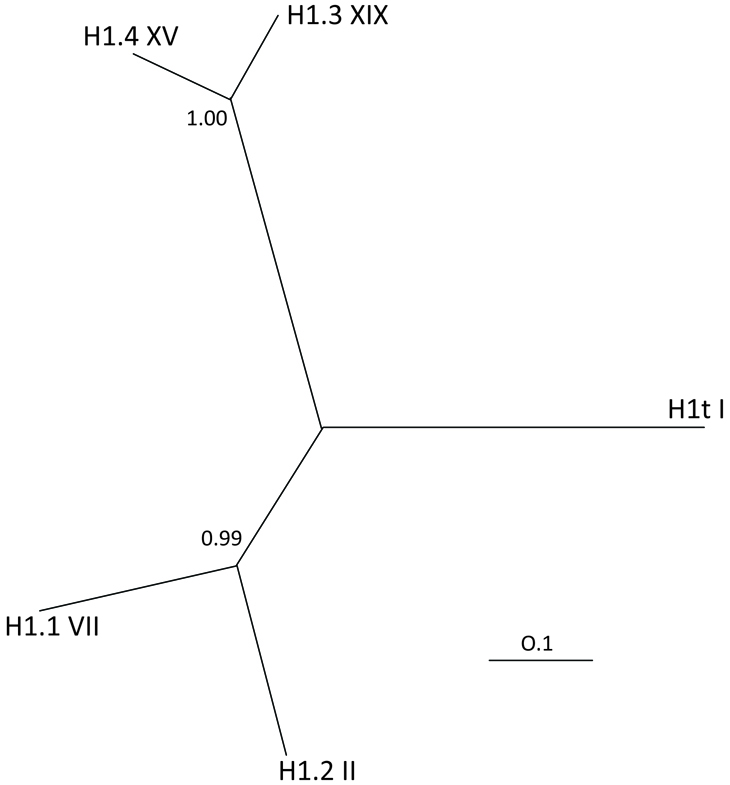


**H4**

**H2B**

**H2A**

**H1**

**H3**

**Figure S1. Phylogenetic relationships among *Oikopleura dioica* histone genes**. Genomic histone sequences including 100 nt of the 5`- and 3`UTR were aligned using PRANK [S1]. PRANK did not always align exons correctly, especially for histone families where only some genes contain introns. Alignments were therefore corrected according to the secondary structure of histone proteins, i.e. the start and stop of alpha helices and loops. Alignments were edited using SeaView [S2] and their reliability was evaluated using GUIDANCE [S3]. A statistical selection of the best-fitting model of nucleotide substitution was carried out with jModeltest [S4]. Subsequently, maximum likelihood phylogenies were computed with PhyML (v3.0.1) [S5]. Trees were reconstructed by maximum likelihood using TREE-PUZZLE [S6]. The chosen models and alpha parameter of the gamma distribution applied to generate trees were:

| **Histone type** | **Selected Model** | **Alpha parameter of gamma distribution** |
| --- | --- | --- |
| H3 | TrN+G | 0.4280 |
| H4 | GTR+G | 0.3950 |
| H2A | HKY+G | 0.7030 |
| H2B | GTR+G | 0.5270 |
| H1 | TrN+G | 1.6050 |

GUIDANCE-results may be viewed at the following links:

Alignment of the H3 histone family:

<http://guidance.tau.ac.il/results/13075250949643/output.php>

Alignment of the H4 histone family:

http://guidance.tau.ac.il/results/13077132239032/output.php

Alignment of the H2B histone family:

http://guidance.tau.ac.il/results/13077132674670/output.php

Alignment of the H2A histone family:

http://guidance.tau.ac.il/results/13077304568012/output.php

Alignment of the H1 histone family:

<http://guidance.tau.ac.il/results/13090307791745/output.php>


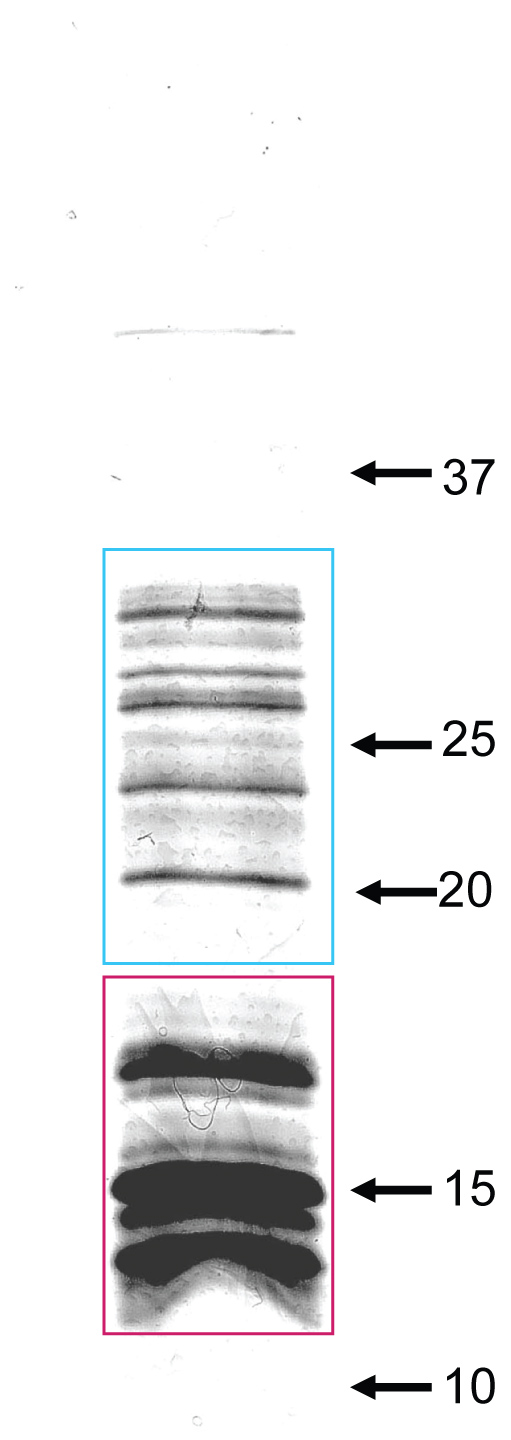


**Figure S2**. **Histones extracted from D4 and D6 animals**. Histones extracted from mixed D4 and D6 animals were loaded on a 15% SDS-PAGE gel and visualized by silver staining. Predicted sizes of protein bands according to molecular weight markers are indicated as arrows in kDa. The main histone bands ran between 12 – 18 kDa. Two different gel slices between 10 – 18 kDa (red frame) and above 18 kDa (blue frame) were excised and analysed separately by mass spectrometry.


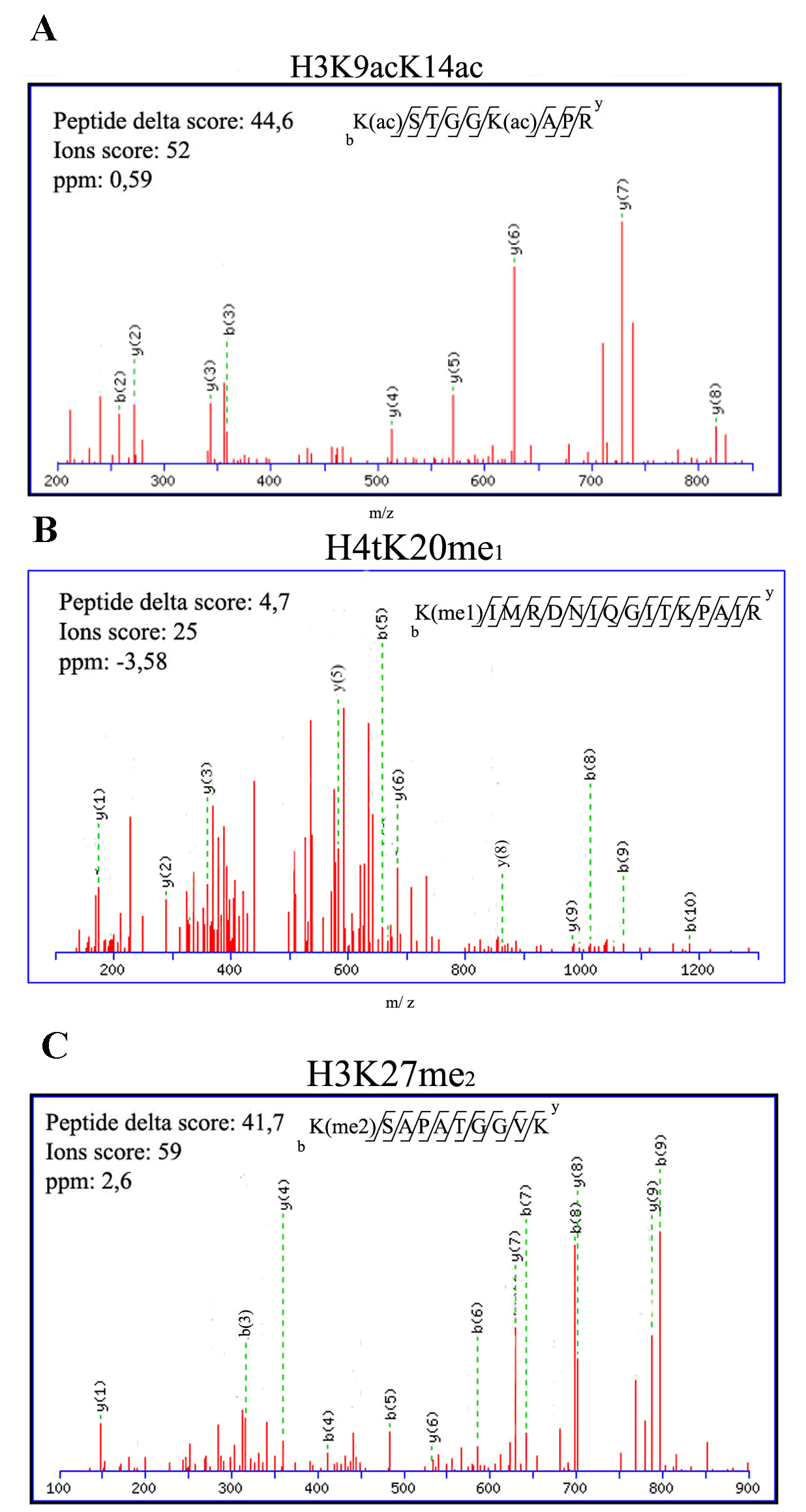


**Figure S3. Spectra of *O. dioica* histone modifications obtained by LC-MS/MS.** The deviation of the parental peptide from predicted mass is given in parts per million (ppm). Only peptides with ppms < ±5 were included in analyses. Ion scores of the parental peptide and the difference between the top match score and the score of the next-best peptide (peptide delta score) are indicated. Examples: A) Doubly acetylated peptide of H3 at K9 and K14. B) Dimethylation of H3 at K27. C) Monomethylation of the male-specific H4t variant at K20. Alternatively H4t could be methylated at R23, resulting in a low delta score.


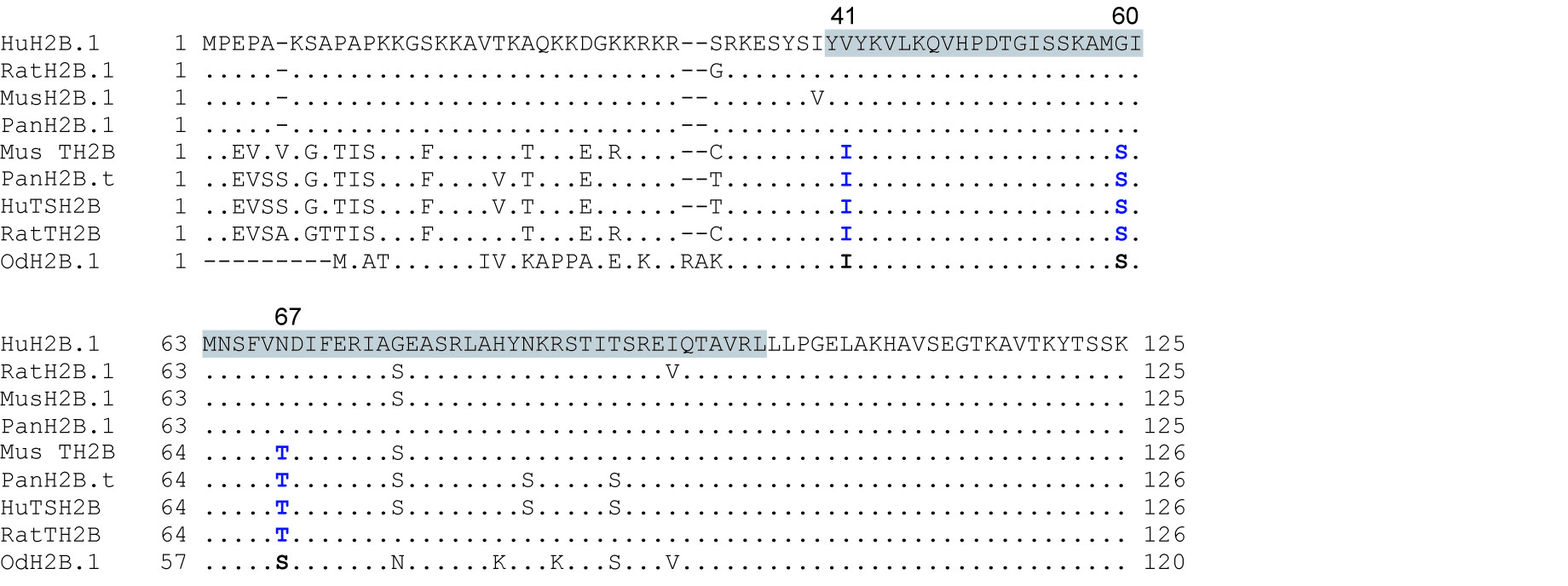


**Figure S4**. **Alignment of mammalian somatic and testes-specific H2B variants with the canonical H2B of *O. dioica***. The testes specific H2B variants of mammals (Hu, human; Rat, rat; Mus, mouse; Pan, chimpanzee) contain three conserved residue changes within the histone fold domain at residue 41, 60 and 67 (dark blue bold). I41 and S60 are present and T67 shows a conservative change to S in all H2B variants of *O. dioica*, including the canonical H2Bs (H2B.1 and H2B.2), which therefore showed higher sequence similarity to testes- rather than somatic H2Bs of mammals.

H3.2 50 EIRRYQKSTELLIRKLPFQRLVREIAQDFKTDLRFQSSAVMALQEASEAYLVGLFED 106

H3.3 50 .....................................A.IG................ 106

OdH3t.1 50 ........................L..QW.S.......................... 106

OdH3t.2 50 ...........................QW.S.......................... 106

OdH3t.3 50 ............L..............QWRN...............A.......... 106

AtMGH3 51 ...K.....D....................V......H..L.....A.......... 107

PfH3.2 50 .........D..................Y.................A.......... 106

PfH3.3 50 ...KS....D.................EY........Q..L.....A.......... 106

Os_465456.1 39 ...K...N...................H..H.M....H..L.....A.......... 95

Os_465459.1 29 ...K...N...................L..H.M....H..L.....A.......... 85

**Figure S5. Histone H3 isoforms of species with substitutions surrounding the methylation site at K79**. Alignment of H3 isoforms identified in *Plasmodium falciparum* (Pf), *Oryza sativa* (Os), *Oikopleura dioica* (Od) and *Arabidopsis thaliana* (At) containing residue substitutions surrounding K79 (green highlight). The partial sequences are aligned to the H3.3 and H3.2 sequences of chordates. Names in blue indicate isoforms exclusive to the male germline.

**Additional Data References**

S1. Löytynoja A, Goldman N: **webPRANK: a phylogeny-aware multiple sequence aligner with interactive alignment browser**. *BMC Bioinformatics* 2010, **11**:1471-2105.

S2. Gouy M, Guindon S, Gascuel O**: SeaView version 4 : A multiplatform graphical user interface for sequence alignment and phylogenetic tree building**. *Mol Biol Evol* 2010, **27**:221-224.

S3. Penn O, Privman E, Ashkenazy H, Landan G, Graur D, Pupko T: **GUIDANCE: a web server for assessing alignment confidence scores**. *Nucl Acids Res* 2010, **38**: 23-28.

S4. Posada D: **jModelTest: Phylogenetic Model Averaging**. *Mol Biol Evol* 2008, **25**:1253-1256.

S5. Guindon S, Gascuel O: (2003) **A simple, fast, and accurate algorithm to estimate large phylogenies by maximum likelihood**. Systematic Biol 2003, **52**:696-704.

S6. **Treepuzzle.** [[http://www.tree-puzzle.de/]](http://www.tree-puzzle.de/%5D).
